# Beta-blocker treatment in the critically ill: a systematic review and meta-analysis

**DOI:** 10.1080/07853890.2022.2098376

**Published:** 2022-07-15

**Authors:** Maria Heliste, Ville Pettilä, David Berger, Stephan M. Jakob, Erika Wilkman

**Affiliations:** a Department of Anesthesiology, Intensive Care and Pain Medicine, University of Helsinki and Helsinki University Hospital, Helsinki, Finland; b Department of Intensive Care Medicine, Inselspital, Bern University Hospital, University of Bern, Bern, Switzerland

**Keywords:** beta-blockers, critically ill, sepsis, trauma, major burns, circulatory shock, intensive care, mortality, systematic review, meta-analysis

## Abstract

**Background:**

Critical illness may lead to activation of the sympathetic system. The sympathetic stimulation may be further increased by exogenous catecholamines, such as vasopressors and inotropes. Excessive adrenergic stress has been associated with organ dysfunction and higher mortality. *β*-Blockers may reduce the adrenergic burden, but they may also compromise perfusion to vital organs thus worsening organ dysfunction. To assess the effect of treatment with *β*-blockers in critically ill adults, we conducted a systematic review and meta-analysis of randomized controlled trials.

**Materials and methods:**

We conducted a search from three major databases: Ovid Medline, the Cochrane Central Register for Controlled Trials and Scopus database. Two independent reviewers screened, selected, and assessed the included articles according to prespecified eligibility criteria. We assessed risk of bias of eligible articles according to the Cochrane guidelines. Quality of evidence was assessed using the Grading of Recommendations Assessment, Development and Evaluation (GRADE) approach.

**Results:**

Sixteen randomized controlled trials comprising 2410 critically ill patients were included in the final review. A meta-analysis of 11 trials including 2103 patients showed a significant reduction in mortality in patients treated with *β*-blockers compared to control (risk ratio 0.65, 95%CI 0.53–0.79; *p* < .0001). There was no significant difference in mean arterial pressure or vasopressor load. Quality of life, biventricular ejection fraction, blood lactate levels, cardiac biomarkers and mitochondrial function could not be included in meta-analysis due to heterogenous reporting of outcomes.

**Conclusions:**

In this systematic review we found that *β*-blocker treatment reduced mortality in critical illness. Use of *β*-blockers in critical illness thus appears safe after initial hemodynamic stabilization. High-quality RCT’s are needed to answer the questions concerning optimal target group of patients, timing of *β*-blocker treatment, choice of *β*-blocker, and choice of physiological and hemodynamic parameters to target during *β*-blocker treatment in critical illness.

**KEY MESSAGES**
- A potential outcome benefit of *β*-blocker treatment in critical illness exists according to the current review and meta-analysis. Administration of *β*-blockers to resuscitated patients in the ICU seems safe in terms of hemodynamic stability and outcome, even during concomitant vasopressor administration. However, further studies, preferably large RCTs on *β*-blocker treatment in the critically ill are needed to answer the questions concerning timing and choice of *β*-blocker, patient selection, and optimal hemodynamic targets.

## Introduction

Critical illness, such as sepsis, trauma and burn injury, promote complex hemodynamic, immunomodulatory, metabolic and coagulation changes. To restore homeostasis, endogenous catecholamines are released, often leading to a sympathetic overstimulation with high concentrations of endogenous catecholamines [1–4]. Exogenous catecholamines may also increase the overall catecholamine load. High levels of both endogenous and exogenous catecholamines are related to organ damage, worse outcome and higher mortality [4–7], independent of blood pressure [8].

The rationale behind the use of *β*-blockers in critically ill is to attenuate persistent high adrenergic stress. Despite high circulating catecholamine levels in shock, adrenergic stimulation is blunted because of alterations at different levels of the signalling pathway, possibly due to the dysfunction of the autonomous nervous system [9,10]. Such dysregulation predicts higher mortality in critically ill patients with multiple organ dysfunction [11]. One of the signs of dysregulation of autonomous nervous system is persistent tachycardia even after treating for other confounding causes for tachycardia, such as hypovolemia and pain. Higher heart rate is independently associated with higher mortality in critical illness [12,13].

*β*-Blockers have been shown to reduce mortality in myocardial infarction (MI) or chronic heart failure (CHF) by reducing heart rate and blood pressure, thus reducing ischaemia, improving ventricular function, and reducing the risk of dysrhythmias, such as atrial fibrillation (AF), which is also a known risk factor for worse outcome in critical illness [14,15].

Concerns regarding the administration of *β*-blockers to critically ill patients exist. By reducing heart rate and lowering arterial pressure, *β*-blockers may compromise cardiac output and organ perfusion, potentially leading to worse outcome. Thus, *β*-adrenergic blockade therapy in critical illness remains controversial. Administration of *β*-blockers with concomitant catecholamine treatment does, however, not seem to compromise hemodynamics, when given to stabilized sepsis patients [16–18]. Moreover, *β*-blockers may restore the sepsis-induced downregulation of adrenergic receptors and suppress the proinflammatory pathways [19,20]. Clinical trials have shown some benefit from *β*-blockers in sepsis, in terms of improved cardiac function, reduced myocardial injury and lower mortality [16,21,22]. In addition, lower mortality and reduced muscle catabolism have been shown in patients with brain injury, general trauma, and burns [23–28]. Previous studies also indicate a potential mortality benefit from *β*-blocker administration prior to critical illness and hospital admission as well as from continuation of *β*-blocker treatment during hospital admission and critical illness [29–34].

We aimed to study whether the use of *β*-blockers is beneficial in critical illness in adults treated in the ICU. To our knowledge, no systematic reviews concerning the use of *β*-blockers in critically ill patients in general exist. We conducted a systematic review of literature and a meta-analysis of randomized controlled trials (RCT) in critically ill adults.

## Materials and methods

### Protocol and registration

The study protocol was registered with the International Prospective Register of Systematic Reviews (PROSPERO; registration number CRD42016050194). The planning and reporting of this review were done according to the Preferred Reporting Items for Systematic Reviews and Meta-analyses (PRISMA) guidelines [35], the Cochrane Handbook for Systematic Reviews of Interventions [36], the University of York’s Centre for Reviews and Dissemination (CRD) guidebook [37] and the Grading of Recommendations Assessment, Development and Evaluation (GRADE) approach [38].

### Eligibility criteria

**Figure 1.**
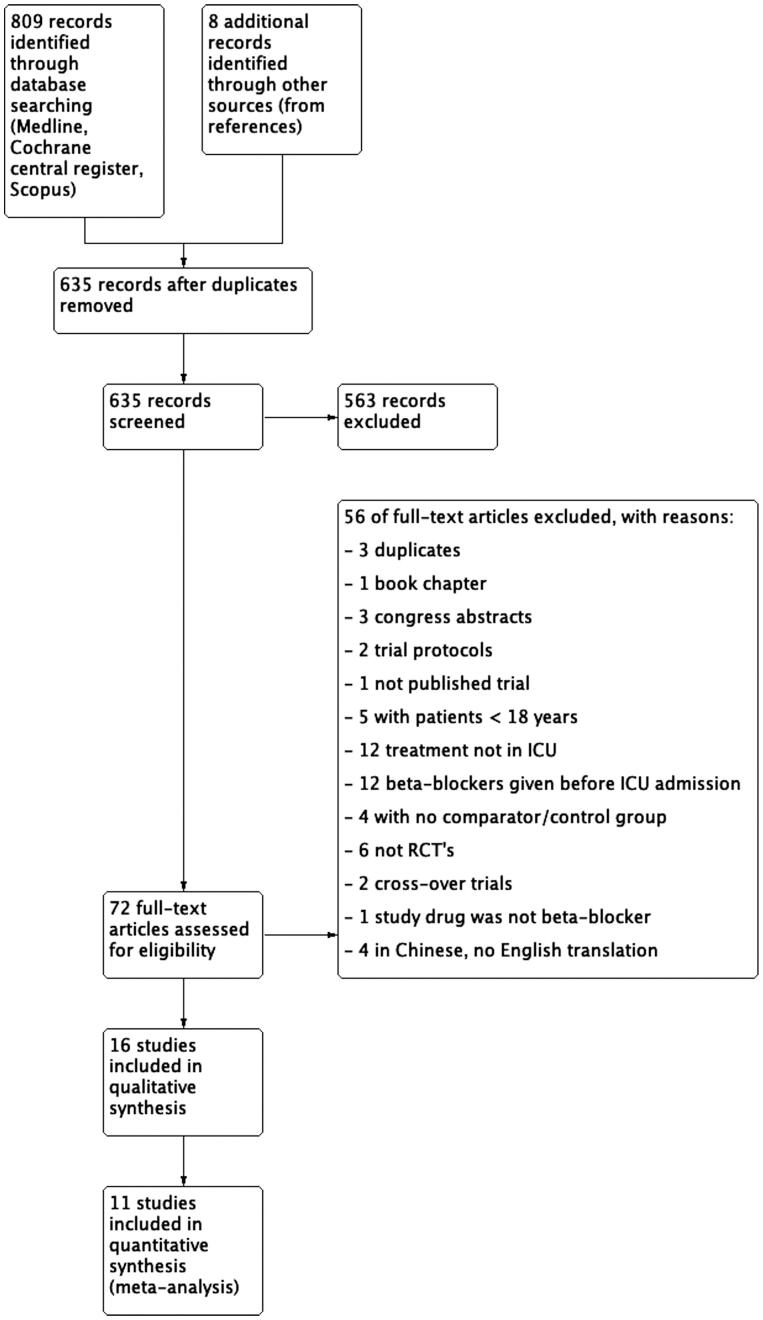
PRISMA flow diagram of the study selection process.

We included randomized controlled trials. Observational and cross-over studies were excluded as well as case reports. The PICO approach (patients (P), intervention (I), control (C) and outcomes (O)) was used for study selection, data extraction, analysis, and synthesis. We included studies comprising adult patients (> 18 years old) treated in the ICU. We excluded studies in which less than 75% of patients were treated in the ICU. The intervention of interest was initiation of *β*-blocker therapy by any route, dose, or frequency in the ICU. We excluded studies in which *β*-blockers had been started before the ICU admission and studies examining the continuation vs. discontinuation of pre-existing *β*-blocker therapy. We included only studies that included a comparator or control group of patients that did not receive *β*-blocker with or without receiving placebo. In case of multiple publications of the same study, articles were handled as a single study. Figure 1 describes the study selection process.

The review question was: How does treatment with *β*-blockers, as compared to placebo or no *β*-blocker, affect patient centred clinical outcomes, physiological outcomes, and organ dysfunction biomarkers in critically ill adults. The main outcomes were mortality at 28/30 days and at 90 days, organ dysfunction, and quality of life. The secondary outcomes were heart rate (HR), mean arterial pressure (MAP), vasopressor load, left or right ventricle ejection fraction (LV/RV EF), presence of diastolic dysfunction, troponin T/I (TnT/I), brain natriuretic peptide (BNP), lactate, changes in mitochondrial function, changes in interleukin 6 (IL-6) and interleukin 10 (IL-10).

### Information sources

We searched three major databases, Ovid Medline, the Cochrane Central Register of Controlled Trials, and Scopus database. We did not apply any restrictions regarding publication date. We also searched for unpublished or ongoing studies from the ClinicalTrials.gov registry and PROSPERO for recently completed systematic reviews. Reference lists of relevant reviews and included trials as well as personal files were reviewed manually for additional publications. We also searched the latest volumes of key journals to find suitable references. The last update for the search was 4.3.2021.

### Search strategy

We included sepsis or septic shock, any kind of circulatory failure, major burns (≥ 30% of total body surface area, TBSA), major trauma and TBI under the term “critical illness”. We did not exclude patients undergoing elective surgery if they were treated postoperatively in the ICU and if the studies met the other eligibility criteria. The search strategy is described in detail in the supplementary material (SM) 1.

### Study selection

Two independent authors (MH and EW) screened the titles and abstracts of the 817 identified articles. 72 potentially relevant trials were obtained in full-text and assessed according to the pre-defined eligibility criteria. Discrepancies were resolved by discussion and in the case of disagreement by a third author (VP). A total for 16 studies were included in the final review. Full-text exclusion reasons are described in detail in SM 2.

### Data extraction

The data extraction process was performed by one author (MH) and checked by another author (EW). Disagreements were resolved by discussion. Extracted data were collected using a predefined data collection form and included following items: study information such as title, first author, country, type of trial (single- vs. multicenter), relevant baseline patient data (numbers of screened and included patients, numbers lost to follow-up/withdrawn, demographic data, type of critical illness), intervention and comparator (if there was one, name of the drug or placebo), starting and duration of treatment, dosage of *β*-blocker and comparator, results of predefined outcomes (organ failure, mortality, quality of life and data concerning hemodynamics and biomarkers), and data concerning methodological quality such as information about randomization process.

### Risk of bias assessment

To assess the internal validity of the included RCT’s we used the Cochrane Collaboration’s tool for assessing risk of bias (RoB) [39]. According to the tool we evaluated each study for its sequence generation, allocation concealment, blinding, appropriateness of outcome measure, incomplete outcome data, and selective outcome reporting. Other bias such as baseline imbalance and reporting of other drugs that affect hemodynamics were also evaluated. The judgement was made independently by two review authors (MH, EW). We rated the risk of bias for each study as “high risk”, “low risk” or “unclear” according to the risk assessment tool. The data needed for assessing risk of bias were collected using the data extraction form. To reduce the publication bias we included only RCT’s. Selective outcome reporting bias was handled by checking the protocols and methods of included studies and compared them to the reported outcomes when possible. The quality of evidence for all outcomes was assessed using the GRADE approach [38] across the domains of risk of bias. A comprehensive description of the assessment of bias is summarized in SM 3.

### Summary measures

We calculated Risk ratios (RR) with 95% confidence intervals (CI) for dichotomous outcomes. For continuous outcomes we calculated mean differences (MD) with 95% CI’s. Analyses were done when at least two studies were available regarding the outcome. All analyses were done using Review Manager 5.4. for non-Cochrane reviews [40].

### Synthesis of results/Meta-analyses

We included all trials that reported predefined outcomes in the meta-analyses. Pooled effect estimates were calculated with Review Manager 5.4. for non-Cochrane reviews [40]. A *p-*value of 0.05 or less was considered statistically significant. Data not included in meta-analyses are presented descriptively as qualitative analysis and synthesis in SM 4.

Heterogeneity among studies was assessed by visual inspection of the forest plots, the chi-squared ($X^{2}$) test and the inconsistency statistics ($I^{2}$). When $I^{2}$ = 0, we used a fixed-effect meta-analysis. When $I^{2}$was > 50% as a marker of moderate heterogeneity, we used both fixed and random effect models and reported the point estimate closest to no effect or the estimate with the widest CI.

One included trial [21] had three treatment groups: milrinone (M), milrinone-esmolol (ME) and control (C, standard care), none of the study groups received *β*-blocker treatment alone. To minimise the effect of milrinone, we combined M and C groups (M + C) and compared ME group with this combined M + C group for mortality analysis. For the analysis of continuous data, we included data only from groups M and ME.

### Additional analyses

We planned to conduct predefined subgroup analyses to explore the potential sources of heterogeneity as follows: (1) severity of illness (the Simplified Acute Physiology Score, SAPS II points <40 and >40), (2) timing of start of intervention (between 0 and 96 h/after 96 h of ICU admission), (3) Esmolol vs. other *β*-blockers, (4) intravenous vs. oral *β*-blockers, (5) high vs. low risk of bias, (6) infection vs. no infection, (7) postoperative vs. medical, (8) elective vs. emergency admissions, (9) cardiac vs. non-cardiac patients.

### Grading the quality of evidence

The GRADE approach [38] was used to assess the quality of evidence for all outcomes across the domains of risk of bias. We rated our confidence in the effect estimate based on risk of bias (limitations in study design), inconsistency of results, indirectness of evidence, imprecision, and publication bias. According to the GRADE approach the overall certainty of evidence was rated as high, moderate, low, or very low.

## Results

### Study selection

Our search identified a total of 817 references (Figure 1). After duplicates were removed, 635 titles remained, and 563 were further excluded in the title and abstract review. A total of 16 RCTs [16,21,23,41–53] with 2410 patients were included in the final review. A detailed list of exclusion reasons for full texts are provided in the SM 2.

### Study characteristics

All included studies were prospective RCTs and published in English between years 1988 and 2020. According to our prespecified inclusion criteria patients were ≥18 years and all patients were treated in the ICU. We included only studies in which the administration of *β*-blocker had been started in the ICU. The number of patients in the individual studies ranged between 26 and 1000. The drug administration was often poorly reported. When reported, the duration of drug administration varied from hours to days. The administration commencement was often reported incompletely or inadequately. Reported follow-up period ranged from 30 min to 8 months, some trials failed to report it clearly. Route of drug administration was either intravenous or per oral or both. Characteristics of the included studies, interventions and reported outcomes are presented in Tables 1–3.

**Table 1.** Characteristics of included studies.

Author, year [ref.]	Country	Blinding, nr. of study centres	Population [type of critical illness]	Nr. of patients; total [beta-blocker/controls]	Follow-up period
Ali, A. et al. 2015 [41]	USA	Non-blinded, single-centre	Severe burns [burns coverin*g* > 30% of TBSA]	69 (35/34)	NR
Arar, C. et al. 2007 [42]	Turkey	Double-blind, single-centre	Cardiac surgery	120 (40/40/40)	Before extubation to 1 min after extubation in the ICU
Balser, J. R. et al. 1998 [43]	USA	Non-blinded, single-centre	Major non-cardiac surgery	63 (34/30*)	12 h
Bible, L. E. et al. 2014 [44]	USA	Non-blinded, single-centre	Severe trauma	45 (25/20)	30 d or until discharge from the hospital, whichever occurred first
Brunner, M. et al. 2000 [45]	Germany	Double-blind, multicenter	Unstable angina pectoris	116 (59/57)	48 h
Cheema, S. A. et al. 2020 [46]	Pakistan	Non-blinded, single-centre	Burns with 20–40% of TBSA	70 (35/35)	NR
Connolly, S. J. et al. 2003 [47]	Canada	Double-blind, single-centre	Heart surgery	1000 (500/500)	14 days or until hospital discharge
De Hert, S. G. et al. 1988 [48]	Belgium	Double-blind, single-centre	Postop treatment after neurosugical interventions for traumatic injury	30 (15/15)	30 min
Er, F. et al. 2016 [49]	Germany	Single-blind, single-centre	STEMI + successful PCI	101 (50/51)	6 months
Guillory, A. N. Et al. 2017 [50]	USA	Non-blinded, single-centre	Severe burns	26 (16/10)	NR
Hanada, K. et al. 2012 [51]	Japan	Non-blinded, single-centre	AMI patients undergoing primary PCI	96 (47/49)	24 h = acute phas*e* + 6 months
Kakihana, Y. et al. 2020 [52]	Japan	Non-blinded, multicenter	Sepsis [+tachyarrhythmia]	151 (76/75)	28 days
Khalili, H. et al. 2020 [23]	USA	Non-blinded, single-centre	Traumatic brain injury	219 (99/120)**	8 months; during hospital stay + at 6 months
Morelli, A. et al. 2013 [16]	Italy	Single-centre, open-label	Septic shock	154 (77/77)	28 d
Sakaguchi, M. Et al. 2012 [53]	Japan	Non-blinded, single centre	Cardiac surgery; AF after valve surgery	60 (30/30)	72 h
Wang, Z. et al. 2015 [21]	China	Non-blinded, single centre	Severe sepsis	90 (30/30/30)	28 d

*Explanations*:

*1 Subject entered the trial twice, randomized to diltiazem group on both times.

**After randomization formed subgroup of isolated severe TBI without other injuries in which number of patients who received propral: 68 = 44%, and control 86 = 56%

*Abbreviations*: Ref.: reference number; NR: not reported; TBSA: total body surface area; ICU: intensive care unit; STEMI: ST-elevation myocardial infarction; PCI: percutaneous coronary intervention; AMI: acute myocardial infarction; AF: atrial fibrillation.

**Table 2.** Aims of included trials.

Author & year [ref.]	Aim of study
Ali, A. et al. 2015 [41]	To investigate effects of propranolol on the cardiovascular system, perioperative hemodynamics, and wound healing by decreasing baseline HR by 20%.
Arar, C. et al. 2007 [42]	To compare the effects of esmolol and Mg on hemodynamic response in the pre-extubation period in the ICU following CABG surgery.
Balser, J. R. et al. 1998 [43]	To evaluate wheather *β*-blockade is better in conversion of SVT than calcium channel blockers in postoperative patients with SVT (FA, flutter or other atrial tachyarrhythmias).
Bible, L. E. et al. 2014 [44]	To investigate whether propranolol would prevent bone marrow dysfunction in humans following severe injury when administered after the injury.
Brunner, M. et al. 2000 [45]	To investigate the safety and efficacy of oral carvedilol in unstable angina in addition to standardised treatment.
Cheema, S. A. et al. 2020 [46]	To compare the mean duration of wound healing and attenuation of muscle wasting in adult burn patients with propranolol and control group.
Connolly, S. J. et al. 2003 [47]	Whether treatment with p.o. metoprolol immediately after heart surgery reduces hospital length of stay and costs.
De Hert, S. G. et al. 1988 [48]	To investigate the influence of labetalol on arterial blood gas data, pulmonary hemodynamics and pulmonary shunting in patients with neurosurgical treatment for traumatic injury.
Er, F. et al. 2016 [49]	To evaluate the role of esmolol-induced tight sympathetic control in STEMI patients with successful PCI.
Guillory, A. N. et al. 2017 [50]	To determine the appropriate propranolol kinetics and dosing strategy for reducing HR in severely burned adults receiving propranolol every 6 h, every 8 h, and once daily.
Hanada, K. et al. 2012 [51]	To examine the efficacy and safety of early i.v. administration of landiolol in patients with AMI undergoing primary PCI.
Kakihana, Y. et al. 2020 [52]	To investigate the effects of landiolol on HR, mortality, and safety in patients with sepsis related tachyarrhythmias, incl. FA, atrial flutter, and sinus tachycardia, compared with patients who received conventional therapy.
Khalili, H. et al. 2020 [23]	To examine the effects of *β*-blockers on survival and functional outcomes in TBI patients.
Morelli, A. et al. 2013 [16]	To investigate the effects of the short-acting *β*-blocker esmolol in patients with severe septic shock (HR and measured subsequent effects on systemic hemodynamics, organ function, adverse events, and 28-day mortality).
Sakaguchi, M. et al. 2012 [53]	To examine the effects of landiolol hydrochloride on prevention of AF and on hemodynamics in the acute postoperative phase after heart valve surgery.
Wang, Z. et al. 2015 [21]	To assess the effects of esmolol combined with milrinone in patients with severe sepsis.

*Abbreviations*: ref.: reference number; HR: heart rate; Mg: magnesium; ICU: intensive care unit: CABG: coronary artery bypass graft; SVT: supraventricular tachycardia; FA: atrial fibrillation; STEMI: ST-elevation myocardial infarction; PCI: percutaneous coronary intervention; i.v.: intravenous; AMI: acute myocardial infarction; TBI: traumatic brain injury.

**Table 3.** Intervention, comparator and reported outcomes of included trials.

Author, year (ref.)	Name of beta-blocker	Control/comparator	Nr. of patients; total (beta-blocker/controls)	Review outcomes reported and included in meta-analysis	Review outcomes reported *
Ali, A. et al. 2015 [45]	Propranolol	Standard care	69 (35/34)	Mortality (no timepoint)	HR, vasopressor load, lactate
Arar, C. et al. 2007 [46]	Esmolol	(1) Magnesium (2) placebo (saline)	120 (40/40/40)	__	HR, MAP
Balser, J. R. et al. 1998 [47]	Esmolol	Diltiazem	63 (34/30*)	Mortality (in-hospital)	HR, MAP, vasopressor load
Bible, L. E. et al. 2014 [48]	Propranolol	Standard care	45 (25/20)	Mortality (30-d)	Organ dysfunction (ventilator days), HR, BP/MAP, lactate
Brunner, M. et al. 2000 [49]	Carvedilol	Placebo; no description of placebo or administration	116 (59/57)	Mortality (48 h), HR	BP (no MAP), TnT
Cheema, S. A. et al. 2020 [50]	Propranolol	Standard care	70 (35/35)	__	HR
Connolly, S. J. et al. 2003 [51]	Metoprolol	Placebo; no description of placebo or administration	1000 (500/500)	Mortality (in-hospital)	Mechanical ventilation, HR
De Hert, S. G. et al. 1988 [52]	Labetalol	Placebo; isotonic physiologic solution	30 (15/15)	__	HR, MAP
Er, F. et al. 2016 [53]	Esmolol	NaCl 0.9 %	101 (50/51)	Mortality (6 months), HR	Quality of life, LV EF, BNP, TnT
Guillory, A. N. Et al. 2017 [54]	Propranolol	Placebo; no description of placebo or administration	26 (16/10)	__	HR, BP (no MAP)
Hanada, K. et al. 2012 [55]	Landiolol	Standard care	96 (47/49)	Mortality (in-hospital, 6 months)	HR, BP (no MAP), LV EF, BNP
Kakihana, Y. et al. 2020 [56]	Landiolol	Standard care	151 (76/75)	Mortality (28 d), HR, MAP, vasopressor load	Organ dysfunction (kidney function, ventilator-free days); LV EF, lactate, BNP, TnI
Khalili, H. et al. 2020 [27]	Propranolol	Standard care	219 (99/120)**	Mortality (in-hospital)	HR, MAP
Morelli, A. et al. 2013 [20]	Esmolol	Standard care	154 (77/77)	Mortality (28-d)	Organ dysfunction (kidneys, liver, heart), HR, MAP, vasopressor load, TnT, lactate
Sakaguchi, M. Et al. 2012 [57]	Landiolol	Standard care	60 (30/30)	__	HR, BP (no MAP), vasopressor load
Wang, Z. et al. 2015 [25]	Esmolol	(1) Milrinone (2) standard care	90 (30/30/30)	Mortality (28-d), HR, MAP, vasopressor load	Organ dysfunction (kidney, liver), lactate, BNP, TnI, IL-6/-10

*Explanations*: *Review outcomes assessed in original trials but not reported as mean (SD)/reported partly/data not available and could not be included in the quantitative meta-analysis.

– = not reported.

*Abbreviations*: ref.: reference number; HR: heart rate; MAP: mean arterial pressure; BP: blood pressure; TnT/TnI: troponin T/I; NaCl: natrium chloride; LVEF: left ventricular ejection fraction; BNP: brain natriuretic peptide; IL-6/-10 : interleukin-6/-10.

### Risk of bias

**Figure 2.**
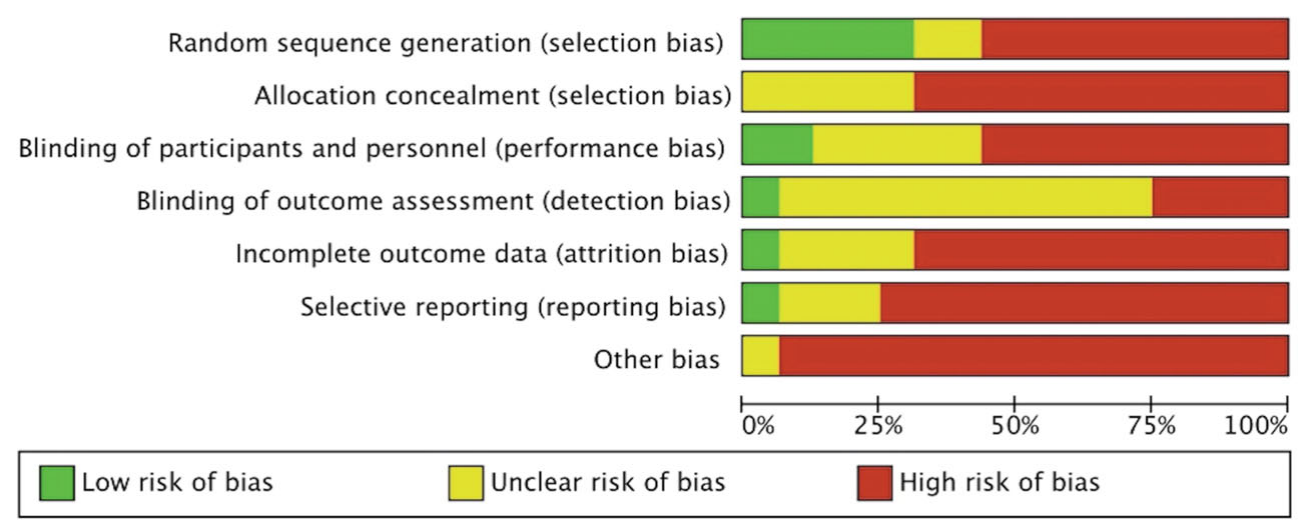
Risk of bias summary.

All included studies were RCT’s. The study protocol was available for four out of 16 trials. Sample size calculations were reported in six and funding in eight out of 16 trials. According to the risk assessment tool [39] none of the included trials were judged as having low overall risk of bias, two trials were judged as having high overall risk of bias and 14 studies were susceptible of having high risk of bias. In the included studies, the domains concerning allocation concealment, incomplete outcome data, selective reporting and other bias were most susceptible to bias. Assessment of risk of bias across studies and risk of bias in individual studies are presented in Figure 2 and SM 3.

### Results of individual studies and data synthesis

The main findings of the included studies are presented in Tables 2 and 3.

We included 16 trials in the qualitative synthesis and 11 trials in the quantitative synthesis. Three studies enrolled sepsis patients [16,21,52], one severe trauma patients [44], three burn patients [41,46,50], two patients with traumatic brain injury [23,48], three cardiological patients (unstable angina pectoris, UAP/ST-elevation myocardial infarction, STEMI/acute myocardial infarction, AMI) [45,49,51], one non-cardiac surgical [43] and three cardiac surgical patients [42,47,53].

The main outcomes were mortality at 28/30 days and at 90 days, organ dysfunction and quality of life at any time point. The secondary/additional outcomes were HR, MAP, vasopressor load, LV/RV EF, presence of diastolic dysfunction, TnT/I, BNP, lactate, changes in mitochondrial function and changes in IL-6/IL-10. According to our prespecified analysis plan, data of secondary outcomes at 48 h after treatment initiation were pooled for meta-analysis if at least two studies were available regarding the outcome. In addition, we conducted meta-analyses also at other timepoints when data were available.

Due to poor reporting of outcomes in original trials, lack of provided information or limited number of trials in each subgroup, we were not able to conduct any of the preplanned subgroup analyses. Also, due to the high risk of bias in most of the included trials, we could not conduct sensitivity analysis based on high- vs. low-risk bias studies.

#### Mortality

Mortality was reported in 11 trials with 2103 patients [16,21,23,41,43–45,47,49,51,52] (Table 3). Three studies enrolled sepsis patients [16,21,52], one severe trauma [44], one burn patients [41], one TBI [23], three cardiological (UAP/STEMI/AMI) [45,49,51], one non-cardiac surgical [43] and one cardiac surgical patients [47]. In-hospital mortality was assessed in four trials [23,43,47,51]. In one trial [45] the follow-up period was short, 48 h, and the reported mortality was therefore likely in-hospital mortality. In one trial, the timepoint of mortality assessment or follow-up period was not reported [41] and there are discrepancies between reported mortality rates [41]. 28- or 30-day mortality was reported in four trials [16,21,44,52]. Two trials [49,51] reported 6-month mortality. Mortality in *β*-blocker group varied between 0 and 49.4% and in control groups between 0 and 80.5%.

In the individual trials, a statistically significant mortality benefit in the *β*-blocker group was reported in three trials. None of the trials reported an increase in mortality with *β*-blocker treatment. Morelli et al. [16] showed a 28-day mortality of 49.4% in the esmolol vs. 80.5% in the control group (*n* = 154, adjusted hazard ratio 0.39, 95% CI 0.26–0.59, *p* < .01) in sepsis patients. In the study by Wang et al. [21] in 90 sepsis patients, overall survival was higher in the combined Milrinone-Esmolol group than in the Milrinone group (Log rank statistic = 5.452; *p* = .020) and control group with standard care (Log rank statistic = 10.206; *p* = .001) group. In the trial of Khalili et al. [23] mortality was significantly lower in a subgroup of patients with isolated severe TBI receiving *β*-blocker treatment compared to patients with isolated severe TBI in the control group (*n* = 154, 4.4 vs. 18.6%, *p* = .012). In their study on *β*-blocker treatment in septic patients, Kakihana et al. [52] showed a lower 28-day mortality rate in patients who met the primary outcome heart rate target of 60–94/min (9 vs. 24%, RR 0.39, 95% CI 0.16–0.92), regardless of treatment group. New-onset arrhythmias were associated with an increase in 28-day mortality (*n* = 151, 46 vs. 11%; RR 4.13 (2.11–8.08)), new-onset arrhythmias were less frequent in the *β*-blocker group (9 vs. 25%, CI 0.15–0.85) *p* = .015.

**Figure 3.**
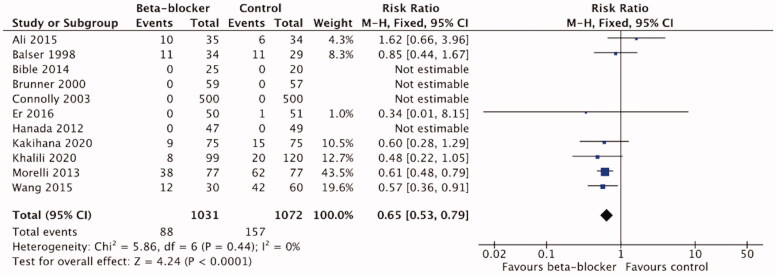
Forest plot of mortality, all.

The meta-analysis of 11 trials [16,21,23,41,43–45,47,49,51,52] in 2103 patients showed a significant reduction in mortality with *β*-blocker treatment compared to the control/standard care (RR 0.65 (95%CI 0.53–0.79; *p* < .0001; $I^{2}=0\%$) (Figure 3). The certainty of evidence using the GRADE approach was high (Table 4).

**Table 4.** GRADE summary of findings table.

Certainty assessment							Summary of findings				
Participants (studies) Follow-up	Risk of bias	Inconsistency	Indirectness	Imprecision	Publication bias	Overall certainty of evidence	Study event rates (%)		Relative effect (95% CI)	Anticipated absolute effects	
							With control	With Beta-blocker		Risk with control	Risk difference with Beta-blocker
Mortality (all)											
2103 (11 RCTs)	Not serious^a^	Not serious	Not serious	Not serious	None	⨁⨁⨁⨁ High	157/1072 (14.6%)	88/1031 (8.5%)	RR 0.65 (0.53 to 0.79)	146 per 1 000	51 fewer per 1 000 (from 69 fewer to 31 fewer)
Short-term mortality (<14 days)											
1467 (5 RCTs)	Not serious	Serious^b^	Not serious	Not serious	None	⨁⨁⨁◯ Moderate	37/740 (5.0%)	29/727 (4.0%)	RR 0.85 (0.45 to 1.60)	50 per 1 000	8 fewer per 1 000 (from 28 fewer to 30 more)
Long-term mortality (>14 days)											
636 (6 RCTs)	Not serious	Not serious	Not serious	Not serious	None	⨁⨁⨁⨁ High	120/332 (36.1%)	59/304 (19.4%)	RR 0.60 (0.48 to 0.74)	361 per 1 000	145 fewer per 1 000 (from 188 fewer to 94 fewer)
Heart rate 24 h (beats/min)											
426 (4 RCTs)	Not serious	Serious^c^	Not serious	Not serious	None	⨁⨁⨁◯ Moderate			*–*		MD 11.96 lower (20.86 lower to 3.06 lower)
MAP 48 h (mmHg)											
210 (2 RCTs)	Not serious	Not serious	Not serious	Not serious	None	⨁⨁⨁⨁ High			*–*		MD 1.66 higher (2.28 lower to 5.61 higher)
MAP 72 h (mmHg)											
210 (2 RCTs)	Not serious	Not serious	Not serious	Not serious	None	⨁⨁⨁⨁ High			*–*		MD 2.43 lower (6.62 lower to 1.75 higher)
Vasopressor load 48 h (µkg/kg/min)											
210 (2 RCTs)	Not serious	Not serious	Not serious	Not serious	None	⨁⨁⨁⨁ High			*–*		MD 0.02 higher (0.02 lower to 0.07 higher)
Vasopressor load 72 h (µkg/kg/min)											
210 (2 RCTs)	Not serious	Not serious	Not serious	Not serious	None	⨁⨁⨁⨁ High			*–*		MD 0 (0.04 lower to 0.03 higher)

CI: confidence interval; MD: mean difference; RR: risk ratio.

*Explanations*: ^a^Despite most trials were considered having high overall-risk of bias, this outcome assessment was considered robust. ^b^Heterogeneity: Tau^2^ = 0.16; Chi^2^ = 4.02, df = 2 (*p* = .13); *I*^2^ = 50%; ^c^Heterogeneity: Tau^2^ = 73.81; Chi^2^ = 32.69, df = 3 (*p* < .00001); *I*^2^ = 91%.

Additionally, we analyzed short-term (<14 days) and long-term (>14 days) mortality rates separately and conducted meta-analyses accordingly. We chose mortality at the longest follow-up reported. In-hospital mortality was included in short-term mortality if the timepoint was not otherwise specified. In one trial [41] the timepoint of mortality or follow-up period were not reported and it was included in the short-term mortality assessment.

**Figure 4.**
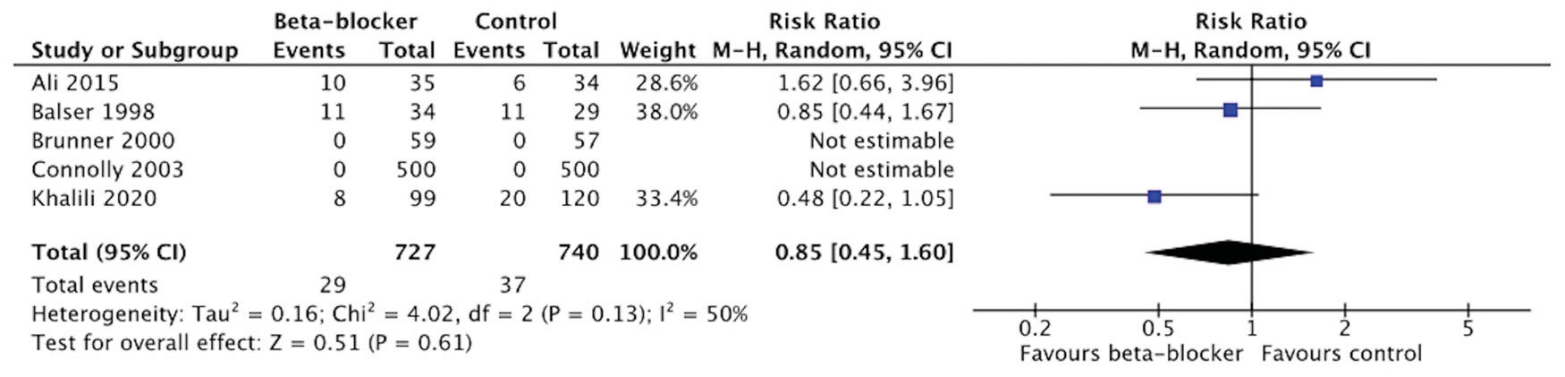
Forest plot of short-term mortality.

Five trials with 1467 patients reported <14-d mortality [23,41,43,45,47], and were included in the short-term mortality meta-analysis. We found no benefit of *β*-blocker treatment on short-term mortality compared to the control/standard care treatment (RR 0.85, 95%CI 0.45–1.60; *p* = .61; $I^{2}=50\%$) (Figure 4). The certainty of evidence using the GRADE approach was moderate (Table 4).

**Figure 5.**
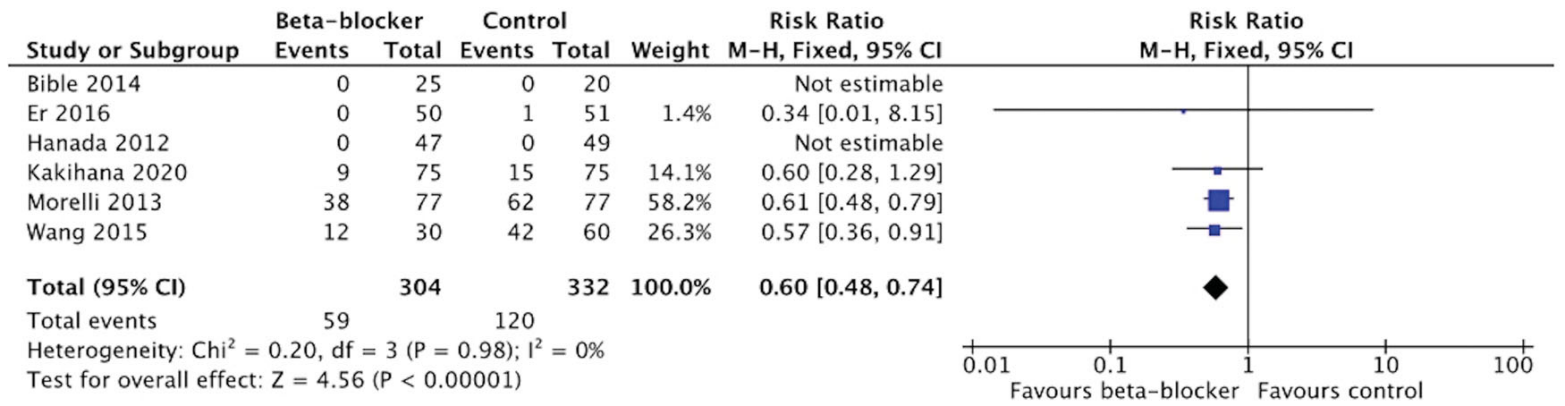
Forest plot of long-term mortality.

Long-term (>14-d) mortality was reported in six trials [16,21,44,49,51,52]) with 636 patients. The meta-analysis showed a significant reduction in long-term mortality with *β*-blocker treatment compared to the control/standard care treatment (RR 0.60, 95%CI 0.48–0.74; *p* < .00001; $I^{2}=0\%$). (Figure 5) The certainty of evidence using the GRADE approach was high (Table 4).

#### Organ dysfunction

Organ dysfunction was reported in five trials [16,21,44,47,52] in patients with severe trauma, heart surgery, and sepsis patients. Data could not be compared and used for meta-analysis due to the varying measurements or reported units. (Table 3)

Mechanical ventilation was assessed in three trials [44,47]: and [52]. In the trial of Bible et al. [44] in severe trauma patients, the propranolol group had numerically less ventilator days (7 vs. 11) (*p* = .97). Connolly et al. [47] reported a significantly increased number of patients in prolonged (>3 days) mechanical ventilation in the metoprolol 6 (1.3%) vs. 0 in the placebo group (*p* = .03) in cardiac surgery patients. In the trial of Kakihana et al. [52] in sepsis patients a total of 101 patients (67%) were mechanically ventilated; of which 57 (75%) were in the landiolol group and 44 (59%) in the control group. Ventilator-free days were similar between the groups (17.0 vs. 17.9, *p* = .47).

Kidney function was assessed in three trials [16]: and [21,52], all with sepsis patients. Kakihana et al. [52] reported kidney function by estimated glomerular filtration rate (eGFR), creatinine and area under the curve (AUC) for eGFR change from baseline to 96 h. AUC for eGFR change from baseline to 96 h was not statistically significant (*p* = .77 in all patients, *p* = .94 in patients without renal replacement therapy (RRT)). No difference in the use of RRT was detected: the landiolol group 23 patients (30%), the control group 24 patients (32%).

Morelli et al. [16] reported better preserved kidney function at 96 h in the esmolol group assessed by median AUC for eGFR (esmolol group 14 mL/min/1.73$m^{2}$(interquartile range, IQR 4–37) vs. control 2 mL/min/1.73$m^{2}$(IQR −7–20; *p* < .001). No difference in RRT during ICU stay, 41.3% in the esmolol group and 41.6% in the control group respectively, were reported.

Wang et al. [21] found a significant improvement of kidney function in Milrinone-Esmolol (ME) group compared to the control group with standard care after 96 h of treatment (*p* < .05). However, this change was reported in both Milrinone (M) and ME groups compared to the control group, but no difference was found between the M and ME groups.

Liver function was assessed in two trials, both in sepsis patients [16,21]. In the trial by Morelli et al. [16] liver function tests did not differ between the groups; no numerical data were presented. In the trial of Wang et al. [21] improvement of liver function at 96 h was reported in the Milrinone-Esmolol (ME) group compared to the control group with standard care (*p* < .05). There was no difference between the ME and Milrinone groups.

#### Quality of life

Quality of life was assessed in one trial [49] but no data were reported.

#### Secondary/additional outcomes

We conducted meta-analyses from HR, MAP, and vasopressor load at 48 and 72 h timepoint. Other data were considerably heterogeneous to be included in the meta-analyses.

#### Heart rate

Heart rate was reported in all 16 included trials (Table 3). Some trials reported data non-numerically, which precluded entry into meta-analyses [16,23,41,43,44,47,48,50,53].

**Figure 6.**
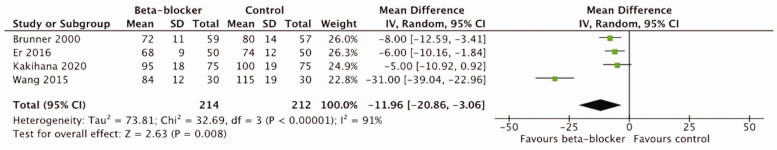
Forest plot of HR 24 h.

HR data as beats/min were reported in seven trials [21,42,45,46,49,51,52]. In 13 trials HR was significantly lower in the *β*-blocker group compared to the control group at some point [16,21,41,42,44–51,53] but the timepoint of measurement varies considerably between the trials or is not reported at all. Four trials (426 patients) [21,45,49,52] reported HR at 24 h and three trials (326 patients) [21,45,52] reported HR at 48 h and were included in the meta-analyses. From the trial of Wang et al. [21] we included the data from milrinone and milrinone-esmolol groups, not the control group with standard care, to minimize the effect of milrinone. There was a significant reduction in HR with *β*-blocker treatment compared to control or standard care at both timepoints. The mean difference (MD) of HR at 24 h was −11.96 (95%CI −20.86 to −3.06), *p =* .008; $I^{2}$=91% and at 48 h MD −13.66 (95%CI −26.10 to −1.22), *p =* .03; $I^{2}$=93%) (Figure 6). The certainty of evidence was moderate (GRADE approach) (Table 4).

Only one trial reported the number of patients with lower vs. higher HR in both groups [52]. Kakihana et al. reported a significantly larger proportion of septic patients in the landiolol group having HR of 60–94/min at 24 h (55%) compared to the control group (33%), (*p* = .0031). In the study by Morelli et al. [16] all patients in the esmolol group achieved the target HR of <95/min compared to the control group (*p* < .001) but the number of patients in the control group was not reported. As only one trial reported the number of patients with lower vs. higher HR in both the intervention and the control group, the data could not be included in a meta-analysis.

#### Mean arterial pressure

**Figure 7.**
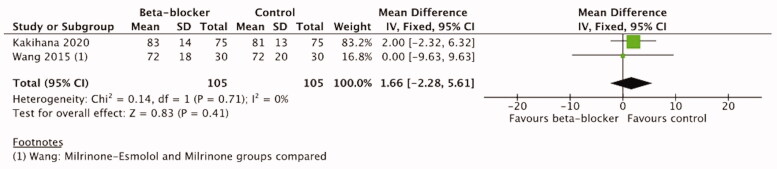
Forest plot of MAP 48 h.

**Figure 8.**
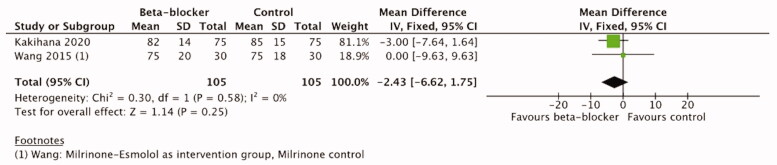
Forest plot of MAP 72 h.

Mean arterial pressure was reported in four trials [21,42,44,52] (Table 3). A single trial of trauma patients [44] reported only one measurement after 2 weeks treatment, the difference between the groups was not statistically significant. One trial of cardiac surgery patients reported numerical data, but the timepoints of measurement are not reported in detail [42]. Two trials in sepsis patients [21,52] reported MAP data at 12, 24, 48, 72, and 96 h after the treatment initiation and were therefore included in the meta-analysis. According to our predefined analysis plan, we included data at 48 h timepoint and in addition at 72 h timepoint. The meta-analysis of these two trials with 210 patients did not show a significant difference between *β*-blocker and control treatment either at 48 h (MD 1.66, 95%CI −2.28–5.61, $I^{2}=0\%,$ *p =* .41) or at 72 h (MD −2.43, 95%CI −6.62–1.75, $I^{2}=0\%,$ *p* = .25). (Figures 7 and 8) The certainty of evidence was high according to the GRADE approach (Table 4).

From the remaining 12 trials [16,23,41,43,45–51,53] data were not reported at all or not could not be included in the meta-analysis (Table 3). None of the trials reported the number of patients with lower vs. higher MAP in the intervention and control groups.

#### Vasopressor load

Vasopressor use was reported in six trials [16,21,41,43,52,53] (Table 3). Two trials did not report numerical data or vasopressor used [41,43]. One trial in cardiac surgery patients [53] reported that the catecholamine dose did not differ between the groups (29 patients (96.7%) in the landiolol group and 28 (93.3%) in the control group). A trial of sepsis patients [16] reported a significant reduction in NA median AUC during the first 96 h in the *β*-blocker group (median AUC relative to baseline value: −0.11 µg/kg/min (IQR −0.46–0) vs. −0.01 µg/kg/min (-0.02–0.44) in the control group, *p* = .003).

Two studies on septic patients reported NA use as µg/kg/min at 12, 24, 48, 72 and 96 h timepoint [21,52]. In the trial by Kakihana et al. [52] there was no difference between the groups as to vasopressor use at any of the trial timepoints. In the trial by Wang et al. [21] all patients were given NA to maintain MAP ≥65 mmHg. They reported a significant decrease in the NA dosage in the Milrinone-Esmolol and Milrinone groups as compared to the control group (0.09 (± 0.8) µg/kg/min and 0.12 (± 0.16) µg/kg/min vs. 0.18 (± 0.14) µg/kg/min, *p* < .05) after 72 h treatment and 0.07 (± 0.04) µg/kg/min and 0.11 (± 0.10) µg/kg/min vs. 0.17 (± 0.12) µg/kg/min, *p* < .05).

**Figure 9.**
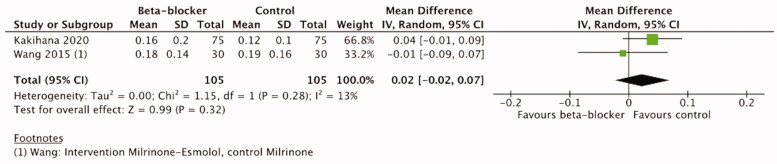
Forest plot of vasopressor load 48 h.

**Figure 10.**
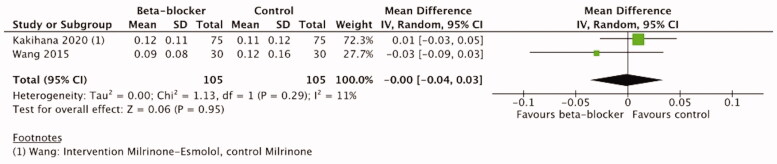
Forest plot of vasopressor load 72 h.

Two hundred ten patients from two trials [21,52] reporting vasopressor load at 48 and 72 h were included in the meta-analysis. From the trial of Wang et al. [21], we included the data from milrinone and milrinone-esmolol groups, not the control group with standard care, to minimize the effect of milrinone. The meta-analysis showed no significant difference between *β*-blocker and control groups either at 48 h (MD 0.02, 95%CI −0.02–0.07, $I^{2}=13\%,\;p=.$32) or at 72 h (MD −0.0, 95%CI −0.04–0.03, $I^{2}=$11%, *p* = .95). (Figures 9 and 10) The certainty of evidence according to the GRADE approach was high (Table 4).

#### Other additional outcomes

We were not able to conduct meta-analyses of left ventricle/right ventricle ejection fraction (LV/RV EF), lactate, BNP, TnI/T, IL-6/IL-10 and mitochondrial function due to the variable measurement timepoints or heterogeneously reported data. These outcomes are presented descriptively as a qualitative analysis and synthesis in SM 4.

## Discussion

### Summary of evidence

In this systematic review of 16 RCT’s, comprising 2410 patients, we found a significant mortality reduction using *β*-blocker treatment compared to placebo or standard care. When analyzing short- and long-term mortality separately, we found a long-term mortality benefit with *β*-blocker treatment compared to placebo or standard care, but no difference in short-term mortality. We also found a significant reduction in HR with *β*-blocker treatment compared to control or standard care at 24 and 48 h timepoints. The meta-analysis did not, however, show a significant difference in MAP between the *β*-blocker and control groups at 48 or 72 h. Nor was there a significant difference in vasopressor load between *β*-blocker and control groups at 48 or 72 h. Data on other outcomes or timepoints were too heterogeneous to be included in the meta-analysis.

#### Previous studies

To our knowledge this is the first systematic review of randomized controlled trials on *β*-blockers in critically ill patients comprising septic, burn, trauma and surgical patients treated in the ICU.

Previously, systematic reviews on *β*-blocker use in septic patients have been published [54–59]. The systematic review by Hasegawa et al. [56] found a significant 28-d mortality benefit with esmolol or landiolol administration in sepsis or septic shock at a risk ratio of 0.68.

Two systematic reviews on RCTs in burn patients have recently been published. These reviews also included studies of burn patients under the age of 18 [60,61]. In these systematic reviews there was no significant differences in mortality between the *β*-blocker and the control groups, but in the individual trials propranolol seemed to improve wound healing. In the current review, only one of the included studies in burn patients provided mortality data [41] and none of the burn trials provided data on other outcomes assessed in the current review. A recent systematic review and meta-analysis in TBI patients [24] showed an in-hospital mortality benefit of *β*-blocker use. The included studies were either observational or retrospective and quality of life or functional outcomes were not assessed.

#### Mortality

In this meta-analysis we found a long-term mortality benefit with *β*-blocker use in critically ill patients. In the individual trials however, significant mortality benefit in *β*-blocker group was reported only in two studies [16,21], both in patients with sepsis. The trial by Morelli et al. has gained attention because of its high mortality in the control group. In the study by Wang et al*. β*-blocker treatment was combined with milrinone in the treatment group. The results by Khalili et al. [23] showed a significantly lower mortality in the subgroup of patients with isolated severe TBI receiving *β*-blockers, but this analysis was performed after data collection, and patients were not originally randomized to that subgroup. Interestingly, Kakihana et al. [52] found a 28-day mortality benefit in patients who met the primary outcome, namely heart rate 60–94/min, regardless of which treatment group the patients were randomized to. The current meta-analysis did not, however, show improvement in short-term (<14 days) mortality. The possible lack of benefit of beta-blockers in short-term mortality may plausibly be explained by differences between individual studies and degree of illness severity. Based on this meta-analysis it is regrettably not possible to draw any conclusions on the observed differences between short-term and long-term mortality. The terms short-term mortality and long-term mortality were used in this review based on the reported follow-up time in the individual studies (<14 days and >14 days). The mortality rates therefore represent different studies and critically ill patient populations. Short-term mortality was reported in only five studies, while long-term mortality was reported in six studies. Only one of the individual studies reported both short- and long-term mortality. Larger randomized controlled studies are needed to deduce possible differences in short- and long-term outcome benefit from beta-blocker treatment in different critically ill patient populations.

#### Heart rate

As tachycardia seems to be an independent risk factor for mortality, heart rate control may be essential for improving survival [12]. Relative bradycardia also associates with lower mortality in patients with septic shock [62]. Worse outcome in patients with tachycardia is possibly due to a shortened diastole and subsequent impairment of diastolic function, leading to worsened coronary perfusion and ischaemia. Moreover, new-onset arrhythmias have been related to greater mortality in critical illness [14,15].

When considering both the underlying physiology, and the results of previous studies mainly including septic patients, there is a rationale for the use of *β*-blockers in critical illness. The optimal target for heart rate control is not known, however. The early-stage compensatory mechanisms of shock may be dependent of a higher heart rate, although some, mainly retrospective studies, have shown benefit of chronic *β*-blockade on survival [30–32,34]. Due to this potentially beneficial compensatory tachycardia, patients included in the RCTs in *β*-blocker use in critical illness have been fluid resuscitated and hemodynamically stabilized before administration of *β*-blocker. A heart rate of 95/min or less has been suggested as target HR for critically ill patients [63] or high risk surgery patients [64]. However, rigid heart rate targets have been criticized, and a rationale for individualized targets based on the state of the patient and stage of illness have been presented [65]. In our review, *β*-blockers caused an expected reduction in heart rate compared to the control/standard care groups. Of note, only two studies targeted HR or presented results on heart rate levels and mortality. Based on the results of these two trials targeting a heart rate of 95/min or below may be beneficial in resuscitated sepsis patients.

#### Blood pressure and organ perfusion

Hypotension and negative inotropy are unfavourable effects of *β*-blockers in critically ill patients. Based on physiological reasoning and earlier clinical trials, however, perfusion may be more important than any given MAP target, bearing in mind that the patients’ needs due to different comorbidities are individual [8]. Previous studies have shown that there is a discrepancy between macro- and micro hemodynamics [66], thus making treatment decisions based on macrohemodynamic parameters potentially misleading. When targeting only a given blood pressure, there is a risk of overzealous vasopressor medication, leading to an increase in catecholamine load and excessive vasoconstriction, which may subsequently worsen microcirculatory perfusion, the improvement of which should be the endpoint of resuscitation. Poor microcirculation in shock has been shown to be a prognostic marker for worse outcome [67]. The downside of this approach is the difficulty in measuring the flow and microcirculation in clinical settings. In this review we found that hemodynamic data were either poorly reported or presented in a low detail.

Based on the current meta-analysis, administration of *β*-blockers to the stabilized patients seems safe, as there were no significant differences in MAP or vasopressor load between the *β*-blocker and control or standard care groups.

#### Choice of beta-blocker

In addition to hemodynamic changes, there are complex immunomodulatory, metabolic and coagulation changes in critical illness. *β*-adrenergic stimulation affects these mechanisms in several ways. Given that *β*-adrenergic effects are complicated and not fully elucidated, it is not clear whether selective or non-selective *β*-blockers should be used, and whether the choice should vary in different critical conditions. Cardioselective *β*-blockers esmolol and landiolol are short-acting, making them a reasonable and attractive choice in the treatment of possibly unstable critically ill patients. In one retrospective study [33], however, non-selective *β*-blockers prior to hospital admission were associated with lower mortality in sepsis patients than cardioselective *β*-blockers. This may indicate an important role of *β*-blockers also in restoring the autonomic nervous homeostasis. The results of another recent retrospective study [31] suggested that in sepsis patients *β*-blockers may have protective effects associated with lowering the heart rate due to resolution of sympathetic overstimulation. Only two individual trials included in the current analysis showed a significant mortality benefit associated with heart rate control, in the trial of Morelli et al. [16] in the *β*-blocker group and in the study by Kakihana et al. [52] in patients with a heart rate below 95 per minute regardless of treatment group. Based on the current review it is not possible to deduce whether the choice of *β*-blocker matters or whether a certain heart rate should be targeted.

Due to considerable heterogeneity and shortcomings of methodology and outcome reporting, conduction of meta-analyses and making conclusions were not always possible. Thus, several outcomes (quality of life, LV/RV EF, lactate, BNP, TnI/TnT and mitochondrial function) could not be included in a meta-analysis. Due to inadequate outcome reporting and variable timepoints or units, comparison of the different studies was cumbersome or even impossible. We conducted meta-analyses when data was provided and statistical tests showed low or moderate heterogeneity, according to the original review protocol. Regrettably, we were not able to conduct any prespecified subgroup or sensitivity analyses.

### Strengths and limitations

All included studies were randomized controlled trials which may be considered the main strength of this review. Inclusion of RCTs only reduces the inherent risk of bias and by excluding retrospective studies publication bias is also reduced. Moreover, by including only RCTs, we were able to follow current guidelines in conducting and reporting a systematic review and meta-analysis, making the methodology firm. Our search yielded RCTs covering a large range of conditions requiring intensive care and to our knowledge, this is the first systematic review on *β*-blockers in critical illness in general.

The main limitation of this study is the lack of large and good quality RCTs in the field. Heterogeneity of measurements, variable reporting of outcomes and poor methodology of the individual trials, render conducting robust and reliable meta-analyses difficult. In addition, lack of reporting of other drugs affecting hemodynamics, e.g. non-study beta-blockers, is a possible source of bias in many of the included trials. The small number of patients included in most studies also make them inherently prone to bias. Lack of published study protocols of the primary studies prohibited the comparison of the study protocol to the published data. In addition, most original trials studied *β*-blockers in patients with sepsis or septic shock, preventing firm conclusions regarding other critical illness states. Of note, three of the original studies included AMI, STEMI and UAP patients [45,49,51], that are not routinely treated in the ICU. To avoid the possible biasing effect of these three trials, we conducted additional sensitivity analyses from which these original trials were excluded (mortality and heart rate at 24 h). The results of the sensitivity analyses did not differ from the original meta-analyses. (ESM 5).

## Conclusions

In this systematic review we found that *β*-blocker treatment reduced long-term mortality in critical illness. No significant differences in MAP or vasopressor load between *β*-blocker treatment and control existed. Administration of *β*-blockers to resuscitated patients in the ICU seems safe in terms of hemodynamic stability and outcome, even during concomitant vasopressor administration.

Further studies, preferably large RCTs on *β*-blocker treatment in the critically ill are needed. Despite the potential outcome benefit shown in this study, many questions, such as those concerning timing and choice of *β*-blocker, patient selection, and optimal hemodynamic targets remain.

## Supplementary Material

Supplemental Material

Supplemental Material

Supplemental Material

Supplemental Material

Supplemental Material

## Data Availability

Data generated or analyzed during this study are included in this published article and its supplementary information files. All background/exctracted data are provided in included articles or their supplementary material available.
