# Supplementary material for: Beta-blocker treatment in the critically ill: a systematic review and meta-analysis
Source: Ann Med. 2022 Jul 15;54(1):1994–2010. doi: 10.1080/07853890.2022.2098376 (PMC9291706; doi:10.1080/07853890.2022.2098376)
Supplement: Supplemental Material [file IANN_A_2098376_SM5525.docx]

**Supplementary material 5. Sensitivity analyses**


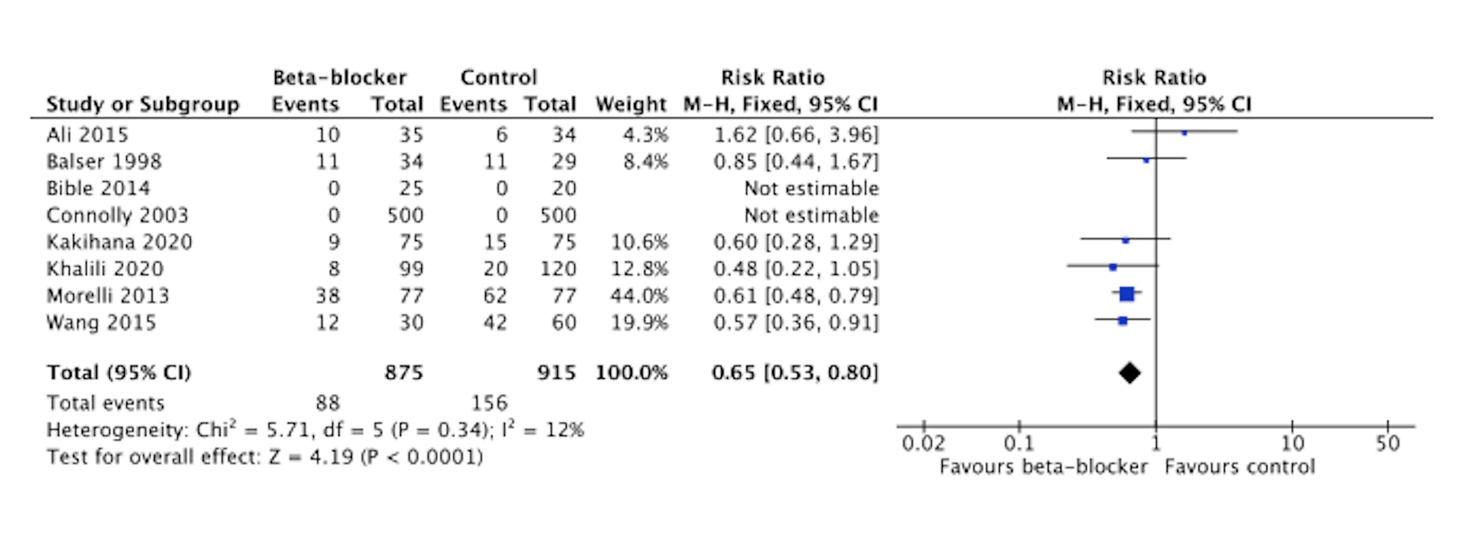
8 trials, 1790 patients; Risk Ratio (95% CI): 0.65 (0.53-0.80); *P*<0.0001

Compared to 11 trials, 2103 patients; Risk ratio (95% CI): 0.65 (0.53-0.79); *P*<0.0001

**Figure 11. Sensitivity analysis for mortality, all (trials of Brunner (45), Er (49) and Hanada (51) removed)**


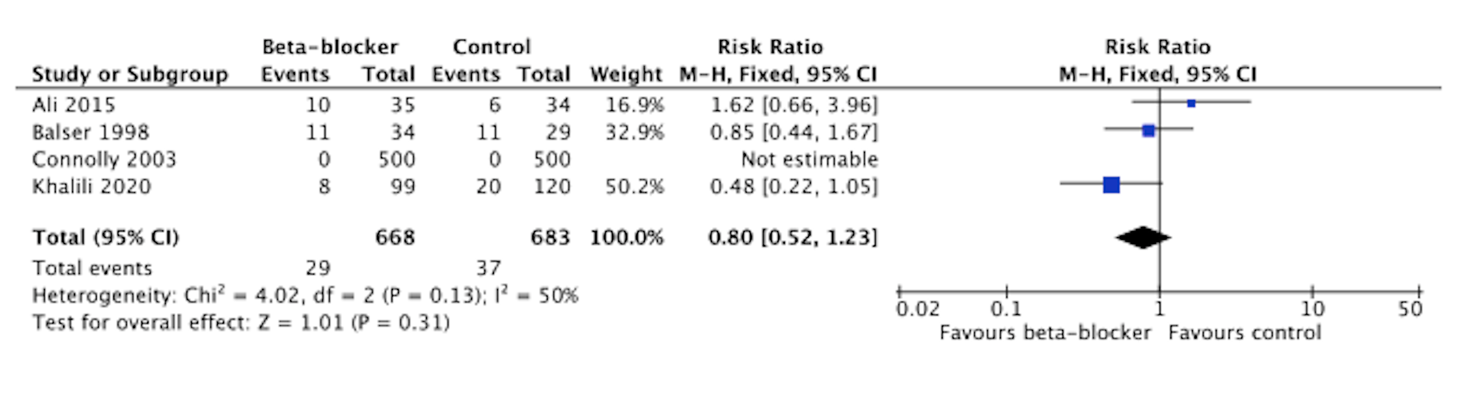


4 trials, 1351 patients; Risk Ratio (95% CI): 0.80 (0.52–1.23); *P*=0.13

Compared to: 5 trials, 1467 patients: Risk Ratio (95% CI): 0.85 (0.45–1.60); *P*=0.61

**Figure 12. Sensitivity analysis for short-term mortality (trials of Brunner (45), Er (49) and Hanada (51) removed)**

**
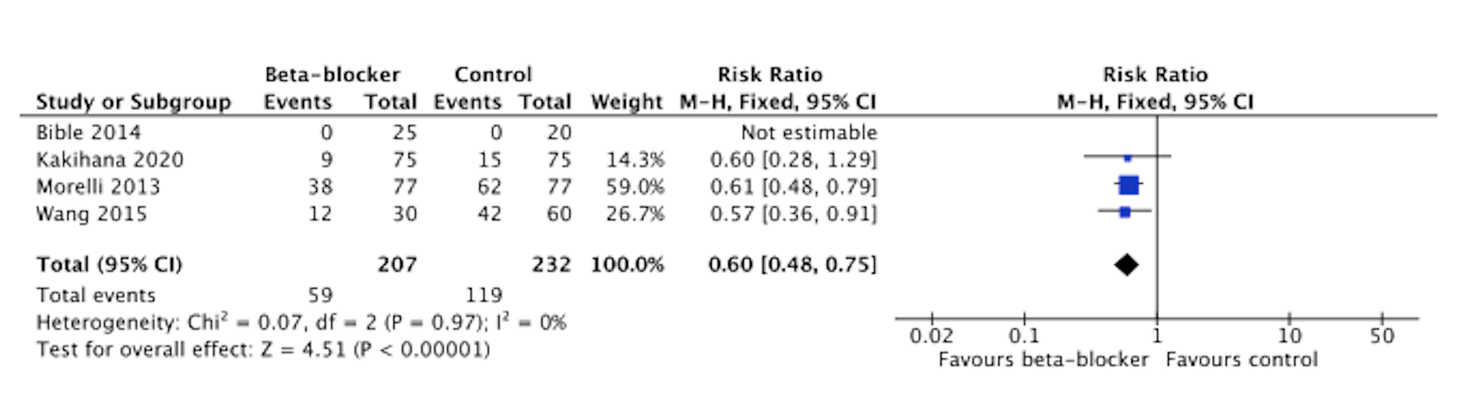
**

4 trials, 439 patients: Risk Ratio (95% CI): 0.60 (0.48–0.75); *P*<0.00001

Compared to: 6 trials, 636 patients: Risk Ratio (95% CI): 0.60 (0.48–0.74); *P*<0.00001

**Figure 13. Sensitivity analysis for long-term mortality (trials of Brunner (45), Er (49) and Hanada (51) removed)**

**
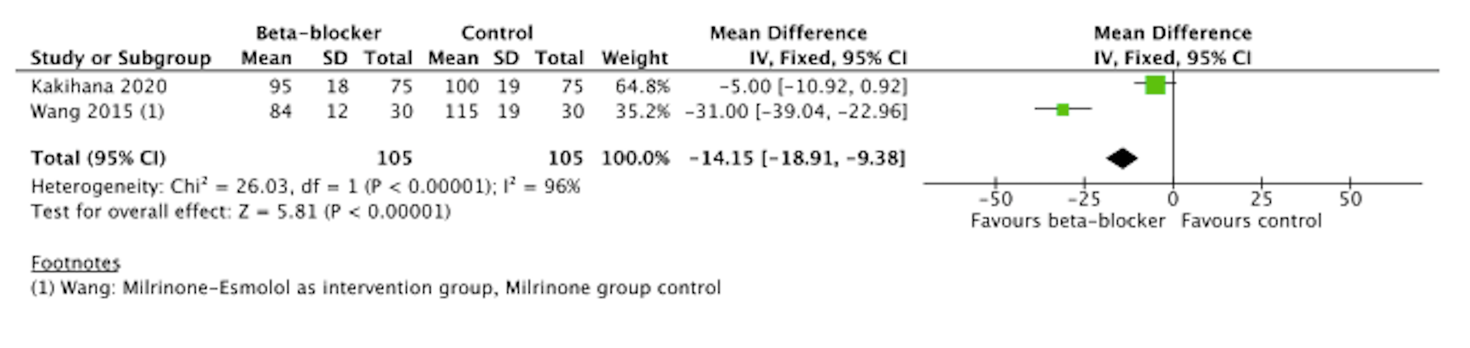
**

2 trials, 210 patients: Risk Ratio (95% CI): -14.15 (-18.91 to -9.38); *P*<0.0001

Compared to: 4 trials, 426 patients: Risk Ratio (95% CI): -11.96 (-20.86 to -3.06); *P*=0.008

**Figure 14. Sensitivity analysis for heart rate 24 h (trials of Brunner (45), Er (49) and Hanada (51) removed)**
