# Supplementary material for: Beta-blocker treatment in the critically ill: a systematic review and meta-analysis
Source: Ann Med. 2022 Jul 15;54(1):1994–2010. doi: 10.1080/07853890.2022.2098376 (PMC9291706; doi:10.1080/07853890.2022.2098376)
Supplement: Supplemental Material [file IANN_A_2098376_SM5524.docx]

**Supplementary material 4. Qualitative analysis and synthesis of the data not included in the meta-analyses.**

**Left/right ventricular ejection fraction**

LV EF was assessed in three trials (1-3) (Table 1-2 below). None of the trials assessed RV EF. Er *et al.* (1) found a statistically significant difference in LV EF in STEMI patients between the esmolol group and the placebo group after 6 weeks (EF 62.5 ± 8.8% in the esmolol group and 58.6 ± 9.3% in the placebo group (*P*=0.035)), but not after 6 months (61.7 ± 9.6% in the esmolol group vs. 60.1 ± 10.1% in the placebo group (*P*=0.407). In the trial of Hanada *et al.* (2) on AMI patients LV EF increased from 49.1 ± 1.5 % in the acute phase (2 weeks after percutaneous coronary intervention (PCI) to 52.0 ± 1.5 % 6 months after PCI (*P*=0.01) in the landiolol group, but no significant difference was found in the control group (from 50.2±1.4% to 50.2 ± 1.2%, *P*=NS). The between-groups difference was not assessed. Kakihana *et al.* (3) assessed LV EF on sepsis patients at an early phase (24–96 h after treatment initiation) but they did not report any differences either within or between the groups.

**Troponin T/I**

Troponin T/I were assessed in five trials (Table 1-2 below). TnT was assessed in three trials of UAP, STEMI and sepsis patients (4),(1),(5), but data were not reported in one trial (4). TnI was assessed in two trials in sepsis patients (3),(6).

In the trial of Er *et al.* (1) on STEMI patients the maximum change in TnT within 48 h was significantly higher in the placebo group (2.5 ng/ml, IQR: 1.0 to 4.0 ng/ml) than in the esmolol group (1.0 ng/ml, IQR: 0.3 to 3.5 ng/ml; *P*=0.010) and the peak TnT was delayed in time in the esmolol group (12 h, IQR: 6–18) compared with the placebo group (6 h; IQR: 6–12; *P*=0.018). Morelli *et al.* (5) also found that markers of myocardial injury in sepsis patients were lower in the esmolol group: the median AUC for TnT in the esmolol group was −0.01 (IQR, −0.05 to 0.00) vs. 0.00 (IQR, −0.01 to 0.02) for the control group (*P*=0.002).

TnI was assessed in two trials of septic patients. Kakihana *et al.* (3) reported no differences in cardiac biomarkers. In the trial of Wang *et al.* (6) significantly lower TnI values were reported in the Milrinone-Esmolol group than in the control and Milrinone groups after 24 h of treatment (*P*<0.05).

**Brain natriuretic peptide**

BNP was assessed in four trials enrolling STEMI, AMI and sepsis patients (1),(2),(3),(6) (Table 1-2 below). Er *et al.* (1) reported lower NT-proBNP values in STEMI patients undergoing primary PCI in the esmolol group compared with the placebo group after 48 hours of treatment initiation (1,048 pg/ml (IQR: 623 to 2,062 pg/ml] vs. 1,497 pg/ml [IQR: 739 to 3,318 pg/ml]; *P*=0.059). Hanada *et al.* (2) did not find a significant difference in BNP values in AMI patients between the esmolol or control group measured on admission (87.5 ±18.8 vs. 77.0±20.8, *P*=0.71) and in chronic phase at 6 months (80.0±11.1 vs. 73.8±13.7, *P*=0.73). Kakihana *et al.* (3) reported no differences in cardiac biomarkers between the landiolol and the control group assessed at 24, 48, 72 and 96 h after treatment initiation. No difference was found between the groups in AUC for the change from baseline to 96 h (esmolol group median 83.9 (IQR-12.9–231.5) vs. control group 50.9 (IQR -41.4–267.7), *P*=0.66). In the trial of Wang *et al.* (6) BNP values were reported significantly lower in the Milrinone-Esmolol group than in the control and Milrinone groups after 24 h of treatment (*P*< 0.05).

**Lactate**

Lactate was measured in five trials (7),(8),(3),(5),(6) (Table 1-2 below). One trial in burn patients did not report numerical data (7) and another trial in trauma patients reported only baseline data (8). Three trials with septic patients (3),(5),(6) reported lactate at 24, 48, 72 and 96 h after treatment initiation but data was not comparable in meta-analysis. Kakihana *et al.* (3) found no difference between the esmolol and the control group in the change from baseline to 96 h (-1.03(2.11)–0.84 (1.46), *P*=0.69). Morelli *et al.* (5) reported that the median AUC for arterial lactate concentration was significantly lower for the esmolol group at −0.1 mmol/L (IQR −0.6 to 0.3) than for the control group at 0.1 mmol/L (IQR, −0.3 to 0.6; *P* =0.006). In the trial of Wang *et al.* (6) lactate was significantly lower in the Esmolol and Milrinone-Esmolol groups at 48, 72 and 96 h after treatment initiation compared to the control group (*P*<0.05).

**Mitochondrial function**

None of the trials assessed changes in mitochondrial function.

**Interleukin 6/10**

Only one trial (6) reported changes in IL-6. Of note, no significant differences among the three groups (Milrinone=M, Milrinone-Esmolol=ME, control=C) existed before treatment, but these measures were significantly lower in the ME group than in C and M groups after 24 h of treatment (*P*<0.05).

Tables 1-2 provided below (please note that the tables and reference numbers differ from the main text article’s tables and ref. numbers).

References (of this supplementary material)

1. Er F, Dahlem KM, Nia AM, Erdmann E, Waltenberger J, Hellmich M, et al. Randomized Control of Sympathetic Drive With Continuous Intravenous Esmolol in Patients With Acute ST-Segment Elevation Myocardial Infarction: The BEtA-Blocker Therapy in Acute Myocardial Infarction (BEAT-AMI) Trial. Jacc: Cardiovascular Interventions. 2016;9(3):231-40.

2. Hanada K, Higuma T, Nishizaki F, Sukekawa T, Yokota T, Yamada M, et al. Randomized study on the efficacy and safety of landiolol, an ultra-short-acting beta1-adrenergic blocker, in patients with acute myocardial infarction undergoing primary percutaneous coronary intervention. Circulation Journal. 2012;76(2):439-45.

3. Kakihana Y, Nishida O, Taniguchi T, Okajima M, Morimatsu H, Ogura H, et al. Efficacy and safety of landiolol, an ultra-short-acting beta1-selective antagonist, for treatment of sepsis-related tachyarrhythmia (J-Land 3S): a multicentre, open-label, randomised controlled trial. Lancet Respir Med. 2020;8(9):863-72.

4. Brunner M, Faber TS, Greve B, Keck A, Schnabel P, Jeron A, et al. Usefulness of carvedilol in unstable angina pectoris. American Journal of Cardiology. 2000;85(10):1173-8.

5. Morelli A, Ertmer C, Westphal M, Rehberg S, Kampmeier T, Ligges S, et al. Effect of heart rate control with esmolol on hemodynamic and clinical outcomes in patients with septic shock: a randomized clinical trial. JAMA. 2013;310(16):1683-91.

6. Wang Z, Wu Q, Nie X, Guo J, Yang C. Combination therapy with milrinone and esmolol for heart protection in patients with severe sepsis: a prospective, randomized trial. Clinical Drug Investigation. 2015;35(11):707-16.

7. Ali A, Herndon DN, Mamachen A, Hasan S, Andersen CR, Grogans RJ, et al. Propranolol attenuates hemorrhage and accelerates wound healing in severely burned adults. Critical Care (London, England). 2015;19:217.

8. Bible LE, Pasupuleti LV, Alzate WD, Gore AV, Song KJ, Sifri ZC, et al. Early propranolol administration to severely injured patients can improve bone marrow dysfunction. The Journal of Trauma and Acute Care Surgery. 2014;77(1):54-60; discussion 59-60.

Table 1 Characteristics of included studies

| Author & year | Country | Population (type of critical illness) | Blinding, nr. of study centers | Nr. of patients; total (beta-blocker/controls) | Name of beta-blocker | Control/  comparator | Follow-up period |
| --- | --- | --- | --- | --- | --- | --- | --- |
| Ali, A. et al. 2015 | USA, Texas | Severe burns (burns covering > 30% of TBSA) | Non-blinded, single-center | 69 (35/34) | Propranolol | Standard care | NR |
| Arar, C. et al. 2007 | Turkey | Cardiac surgery | Double-blind, single-center | 120 (40/40/40) | Esmolol | 1) magnesium 2) placebo (saline) | Before extubation to 1 minute after extubation in the ICU |
| Balser, J. R. et al. 1998 | Maryland, USA | Major non-cardiac surgery | Non-blinded, single-center | 63 (34/30*) | Esmolol | Diltiazem | 12 h |
| Bible, L. E. et al. 2014 | Newark, New Jersey, USA | Severe trauma | Non-blinded, single-center | 45 (25/20) | Propranolol | Standard care | 30 d or until discharge from the hospital, whichever occured first |
| Brunner, M. et al. 2000 | Germany | Unstable angina pectoris | Double-blind, multicenter | 116 (59/57) | Carvedilol | Placebo; no description of placebo or administration | 48 h |
| Cheema, S. A. et al. 2020 | Pakistan | Burns with 20-40% burn of TBSA | Non-blinded, single-center | 70 (35/35) | Propranolol | Standard care | NR |
| Connolly, S. J. et al. 2003 | Ontario, Canada | Heart surgery | Double-blind, single-center | 1000 (500/500) | Metoprolol | Placebo; no description of placebo or administration | 14 days or until hospital discharge |
| De Hert, S. G. et al. 1988 | Belgium | Postop treatment after neurosugical interventions for traumatic injury | Double-blind, single-center | 30 (15/15) | Labetalol | Placebo; isotonic physiologic solution | 30 minutes |
| Er, F. et al. 2016 | Germany | STEMI + successful PCI | Single-blind, single-center | 101 (50/51) | Esmolol | NaCl 0.9% | 6 months |
| Guillory, A. N. et al. 2017 | Texas, USA | Severe burns | Non-blinded, single-center | 26 (16/10) | Propranolol | Placebo; no description of placebo or administration | NR |
| Hanada, K. et al. 2012 | Hirosaki, Japan | AMI patients undergoing primary PCI | Non-blinded, single-center | 96 (47/49) | Landiolol | Standard care | 24 h = acute phase + 6 months |
| Kakihana, Y. et al. 2020 | Japan | Sepsis (+tachyarrhythmia) | Non-blinded, multicenter | 151 (76/75) | Landiolol | Standard care | 28 days |
| Khalili, H. et al. 2020 | Dallas, USA | Traumatic Brain Injury (TBI) | Non-blinded, single-center | 219 (99/120)** | Propranolol | Standard care | 8 months; during hospital stay + at 6 months |
| Morelli, A. et al. 2013 | Rome, Italy | Septic shock | Single-center, open-label | 154 (77/77) | Esmolol | Standard care | 28 d |
| Sakaguchi, M. et al. 2012 | Osaka, Japan | Cardiac surgery; AF after valve surgery | Non-blinded, single center | 60 (30/30) | Landiolol | Standard care | 72 h |
| Wang, Z. et al. 2015 | Nanchang, Jiangsu, China | Severe sepsis | Non-blinded, single center | 90 (30/30/30) | Esmolol | 1) Milrinone 2) standard care | 28 d |

Explanations:

| *1 subject entered the trial twice, randomized to diltiazem group on both times |
| --- |
| ** after randomization formed subgroup of isolated severe TBI in which number of patients who received propral: 68 = 44%, and control 86 = 56% |
| NR = not reported |

Table 2 Aims of studies and reported outcomes

| Author & year | Aim of study | Review outcomes reported and included in meta-analysis | Review outcomes reported * |
| --- | --- | --- | --- |
| Ali, A. et al. 2015 | To investigate effects of propranolol on the cardiovascular system, perioperative hemodynamics and wound healing by decreasing baseline heart rates by 20% | Mortality (no timepoint) | HR, vasopressor load, lactate |
| Arar, C. et al. 2007 | To compare the effects of esmolol and Mg on hemodynamic response in the pre-extubation period in the ICU following CABG surgery | __ | HR, MAP |
| Balser, J. R. et al. 1998 | To evaluate wheather beta blockade is better in conversion of SVT than calcium channel blockers in postoperative patients with SVT (FA, flutter or other atrial tachyarrhythmias) | Mortality (in-hospital) | HR, MAP, vasopressor load |
| Bible, L. E. et al. 2014 | To investigate whether propranolol would prevent bone marrow dysfunction in humans following severe injury when administered after the injury | Mortality (30-d) | Organ dysfunction (ventilator days), HR, BP/MAP, lactate |
| Brunner, M. et al. 2000 | The investigate the safety and efficacy of oral carvedilol in unstable angina in addition to standardized treatment | Mortality (48 h), HR | BP (no MAP), TnT |
| Cheema, S. A. et al. 2020 | To compare the mean duration of wound healing and attenuation of muscle wasting in adult burn patients with propranolol and control group | __ | HR |
| Connolly, S. J. et al. 2003 | Whether treatment with p.o. metoprolol immediately after heart surgery reduces hospital length of stay and cost | Mortality (in-hospital) | Mechanical ventilation, HR |
| De Hert, S. G. et al. 1988 | To investigate the influence of labetalol on arterial blood gas data, pulmonary haemodynamics and pulmonary shunting in patients with neurosurgical treatment for traumatic injury | __ | HR, MAP |
| Er, F. et al. 2016 | To evaluate the role of esmolol-induced tight sympathetic control in STEMI patients with successful PCI | Mortality (6 months), HR | Quality of life, LV EF, BNP, TnT |
| Guillory, A. N. et al. 2017 | To determine the appropriate propranolol kinetics and dosing strategy for reducing HR in severely burned adults receiving propranolol every 6h, every 8h, and once daily | __ | HR, BP (no MAP) |
| Hanada, K. et al. 2012 | To examine the efficacy and safety of early i.v. administration of landiolol in patients with AMI undergoing primary PCI. | Mortality (in-hospital, 6 months) | HR, BP (no MAP), LV EF, BNP |
| Kakihana, Y. et al. 2020 | To investigate the effects of landiolol on HR, mortality, and safety in patients with sepsis­related tachyarrhythmias, incl. atrial fibrillation, atrial flutter, and sinus tachycardia, compared with patients who received conventional therapy. | Mortality (28 d), HR, MAP, vasopressor load | Organ dysfunction (kidney function, ventilator-free days); LV EF, lactate, BNP, TnI |
| Khalili, H. et al. 2020 | To examine the effects of beta-blockers on survival and functional outcomes in TBI patients. | Mortality (in-hospital) | HR, MAP |
| Morelli, A. et al. 2013 | To investigate the effects of the short-acting beta blocker esmolol in patients with severe septic shock (HR and measured subsequent effects on systemic hemodynamics, organ function, adverse events, and 28-day mortality). | Mortality (28-d) | Organ dysfunction (kidneys, liver, heart), HR, MAP, vasopressor load, TnT, lactate |
| Sakaguchi, M. et al. 2012 | To examine the effects of landiolol hydrochloride on prevention of AF and on hemodynamics in the acute postoperative phase after heart valve surgery. | __ | HR, BP (no MAP), vasopressor load |
| Wang, Z. et al. 2015 | To assess the effects of esmolol combined with milrinone in patients with severe sepsis. | Mortality (28-d), HR, MAP, vasopressor load | Organ dysfunction (kidney, liver), lactate, BNP, TnI, IL-6/-10 |

Explanations:

*Review outcomes assessed in original trials but not reported as mean (SD)/reported partly/data not available and could not be included in the quantitative meta-analysis

-- = not reported

Abbreviations:

HR=heart rate, CABG=Coronary Artery Bypass Graft, MAP=mean arterial pressure, Mg=Magnesium, SVT=supraventricular tachycardia, FA=Atrial Fibrillation, BP=Blood pressure, p.o.=per oral, STEMI=ST-elevation myocardial infarction, PCI=percutaneous coronary intervention, LV EF = left ventricular ejection fraction, BNP = brain natriuretic peptide, TnT/TnI = troponin T/I, i.v.=intravenous, AMI= Acute myocardial infarction
