# Supplementary material for: Beta-blocker treatment in the critically ill: a systematic review and meta-analysis
Source: Ann Med. 2022 Jul 15;54(1):1994–2010. doi: 10.1080/07853890.2022.2098376 (PMC9291706; doi:10.1080/07853890.2022.2098376)
Supplement: Supplemental Material [file IANN_A_2098376_SM5523.docx]

Supplementary material 3.

Assessment of risk of bias according to the Cochrane collaborations’s RoB tool. Risk of bias in individual trials, included trials in alphabetical order.


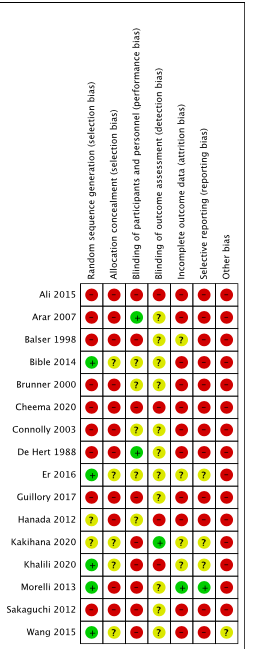


Ali et al.

Arar et al.

Balser et al.

Bible et al.

Brunner et al.

Cheema et al.

Connolly et al.

De Hert et al.

Er et al.

Guillorry et al.

Hanada et al.

Kakihana et al.

Khalili et al.

Morelli et al.

Sakaguchi et al.

Wang et al.
