# Supplementary material for: Beta-blocker treatment in the critically ill: a systematic review and meta-analysis
Source: Ann Med. 2022 Jul 15;54(1):1994–2010. doi: 10.1080/07853890.2022.2098376 (PMC9291706; doi:10.1080/07853890.2022.2098376)
Supplement: Supplemental Material [file IANN_A_2098376_SM5522.docx]

**Supplementary material 2**

**Fulltext exclusion reasons:**

- **3 Duplicates:**
- Balser (1998) duplicate, title with different spelling of “beta blocker”/“β-blocker”
- Cruickshank, J.M. et al (1988): duplicate, similar article from the same writer year published in 1987; different titles and published in different journals. Titles: “Reduction of stress/catecholamine-induced cardiac necrosis by beta 1-selective blockade” published in Lancet in 1987 and “Stress/catecholamine-induced cardiac necrosis. Reduction by beta 1-selective blockade” published in Postgraduate Medicin in year 1988. The 1987 article was excluded because it recruited patients under 18 years old.
- Liu Xinqiang (2015) =duplicate with Xinqiang, Liu (2015)
- **1 book chapter:**
- Mohr, A & Alamo I: Propranolol: A protective mechanism for bone marrow dysfunction following injury.
- **3 Conference abstracts:**
- Fayed, A. et al. (2009)
- Morelli, A. et al. (2011)
- Osada, H. et al. (2012)
- **2 trial protocols:**
- Nct (Evaluation of Early CRRT and Beta-blocker InTerventions in Patients With ECMO(ELITE). (2018)
- Nct (Beta-blocker Before Extubation) (2007)
- **1 not published trial:**
- Isrctn (no author): Safety and efficacy of landiolol in controlling heart rate of supraventricular tachyarrhythmias in patients with severe sepsis – Checked from the Clinicaltrialsgov.: Updated 2013, "results overdue"
- **5 with patients < 18 years old:**
- Bhorat I. et al. (1993)
- Cruickshank, J. M et al. (1987) – also a duplicate with the article from the same writer in year 1988
- Mabie W. et al (1987)
- Mohammadi, A. A. (2009)
- Raczynski, J. et al. (1983)
- **12 in which treatment site is not ICU (e.g. CCU) or treatment site is not clear or population not critically ill:**
- Anonymous (HINT trial group) (1986) – also same trial than Lubsen 1987
- Assmann I. et al. (1981) - All patients with cardiogenic shock or decompensation are excluded - not critically ill, no statement about treatment site + not RCT (?)
- Balcon, R. et al. (1967)
- Balkin, J. et al. (1994) – CCU
- Chen, Z. et al. (2005) - based on avaliable data, assume that all patients have not been treated in the ICU (it is not clearly stated)
- Easterling, T. et al. (2019) – Patients were not treated in the ICU: "we also assessed the duration of hospital stay, admission to an intensive care unit - - " and in table 3. "maternal outcomes" there are 0 admissions from each group
- Harper, A., Murnaghan, G.A. (1991) - also outcome is fetal-centered
- Lubsen, J., Tijssen, J. G. (1987)
- Orrey, D. C. et al. (2015)
- Sathya Lakshmi, B., Dasari, P. (2012) (Also outcome is not patient-centered, The primary outcome was the number of doses required to achieve target BP and time required to reduce the mean arterial pressure by 25%)
- Waagstein F & Hjalmarson AC (1976)
- Wasim, T. et al (2020) – Treatment site is not reported, pregnant women with pre-eclampsia, assumably not all in the ICU
- **12 in which beta blockers given before ICU admission/unclear whether it was started before:**
- Aoki, Y. et al. (2020)
- Behmanesh, S. et al. (2006) - Control group did receive their preop medication incl. BB (82%) - no control group that did not receive BB! In Prospero we state that there has to be a control group that did not receive BB and that BB should be started in the ICU.
- Ciszewski, P. et al. (2013)
- Friese, R. S. et al. (2008) - Randomized intervention (BB) group+observational (preshospital BB) group were combined and handled as a single intervention group – based on this exclude because we state in the Prospero that intervention should be started in the ICU; in this trial there is no group in which BB was started after the admission, because the groups were mixed
- Hallerback, B. et al. (1987)
- Ito, N. et al. (2012)
- Mooss, A. N. et al. (2000)
- Neto, J. M. R. et al. (2013)
- Roolvink, V. et al. (2016)
- Sezai, A. et al. (2011)
- Sezai, A. et al. (2015)
- Varon, J. et al. (2014)
- **4 with no comparator group (other than (another) beta blocker):**
- Abraham, A. S. et al. (1994)
- Chatzidou, S. et al. (2018) - propranolol vs. metoprolol
- Fujii, M. et al. (2012) - i.v. landiolol + p.o. carvedilol more effective than p.o. carvedilol alone - no control without BB
- Iliuţǎ, L. (2004) - BB compared to another BB
- **6 not RCT/not clearly stated as RCT (process not described):**
- Ji, M. et al. (2014)
- Lira, A. & Pinsky, M. (2014) - Journal club, discussion about Morelli's trial
- Shang, X. et al. (2016) (it also seems that patients had esmolol already before dividing into groups)
- Shi, Q. et al. (2015)
- Sweeney, R. M. et al. (2010)
- Van Diepen, S. et al. (2014)
- **2 cross-over trials:**
- Gracia, P.D. et al. 2006
- Natale, E. et al. (1991)
- **1 in which study drug was not beta-blocker as it was reported in (metoprolol in the abstract, later in the article the name of the drug changed to thiazide diuretic “metoral”)**
- Kamali, A. et al. 2017
- **4 in Chinese,** **English translation missing**, published only in Chinese journals
- Liu H. et al. (2019)
- Wang S. et al (2017)
- Xinqiang, Liu (2015) = Liu Xinqiang et al. (2015) (duplicate)
- Yang, S. et al (2014)
