# Supplementary material for: Beta-blocker treatment in the critically ill: a systematic review and meta-analysis
Source: Ann Med. 2022 Jul 15;54(1):1994–2010. doi: 10.1080/07853890.2022.2098376 (PMC9291706; doi:10.1080/07853890.2022.2098376)
Supplement: Supplemental Material [file IANN_A_2098376_SM5510.docx]

**Supplementary material 1**

**Search strategy 9/2019 and update 3/2021; Ovid Medline, Scopus, EBM Reviews - Cochrane Database of Systematic Reviews**

Database: Ovid MEDLINE(R) and Epub Ahead of Print, In-Process & Other Non-Indexed Citations and Daily <1946 to September 13, 2019>

Search Strategy:

--------------------------------------------------------------------------------

1 (critically ill or intensive care or critical illness or critical care).mp. (223848)

2 exp Critical Care/ (55095)

3 exp Critical Illness/ (26495)

4 severely ill.mp. (4346)

5 sepsis.mp. or exp SEPSIS/ (172676)

6 septic shock.mp. or exp Shock, Septic/ (32957)

7 exp SHOCK/ or shock.mp. (229372)

8 circulatory failure.mp. (2068)

9 exp Intensive Care Units/ (78856)

10 emergency department.mp. or exp Emergency Service, Hospital/ (116141)

11 trauma.mp. (252407)

12 exp Trauma Centers/ (9929)

13 exp Multiple Trauma/ (12555)

14 severe injury.mp. (3392)

15 exp "Wounds and Injuries"/ or severely injured.mp. (875698)

16 severe burn.mp. (1741)

17 exp Burns/ (56278)

18 1 or 2 or 3 or 4 or 5 or 6 or 7 or 8 or 9 or 10 or 11 or 12 or 13 or 14 or 15 or 16 or 17 (1590349)

19 beta adrenergic blocker.mp. or exp Adrenergic beta-Antagonists/ (83408)

20 exp adrenergic beta-antagonists/ or exp adrenergic beta-1 receptor antagonists/ or exp adrenergic beta-2 receptor antagonists/ or exp adrenergic beta-3 receptor antagonists/ (83212)

21 landiolol.mp. (333)

22 esmolol.mp. (1412)

23 propranolol.mp. or exp PROPRANOLOL/ (44555)

24 bisoprolol.mp. or exp BISOPROLOL/ (1648)

25 metoprolol.mp. or exp METOPROLOL/ (8057)

26 19 or 20 or 21 or 22 or 23 or 24 or 25 or beta blocker*.mp. (108159)

27 exp Multiple Organ Failure/ or organ failure.mp. (24772)

28 organ dysfunction.mp. (11701)

29 recovery.mp. (456678)

30 27 or 28 or 29 (487981)

**31** 18 and 26 (3508)

32 limit 31 to ("all adult (19 plus years)" and clinical trial, all) (233)

33 exp MORTALITY/ or mortality.mp. (1193477)

34 outcome.mp. or exp Treatment Outcome/ or exp "OUTCOME ASSESSMENT (HEALTH CARE)"/ (1859631)

35 exp SURVIVAL/ or survival.mp. (1168440)

36 30 or 33 or 34 or 35 (3671106)

37 18 and 26 and 36 (1404)

38 limit 37 to ("all adult (19 plus years)" and clinical trial, all) (132)

39 beta blocker*.mp. (29546)

40 limit 32 to yr="2019 -Current" (1)

41 (clinical or randomized or RCT).af. (5813862)

42 31 and 41 (1399)

43 (adult* or aged or elderly).af. (7792055)

44 42 and 43 (794)

45 limit 44 to yr="2019 -Current" (8)

**SCOPUS**

**( ( TITLE-ABS-KEY ( "critically ill" OR "intensive care" OR "critical illness" OR "critical care" ) ) AND ( ( TITLE-ABS-KEY ( "beta adrenergic blocker" OR "beta blocker" ) OR TITLE-ABS-KEY ( esmolol OR landiolol OR bisoprolol OR metoprolol OR labetalol ) ) ) AND ( TITLE-ABS-KEY ( randomized OR rct ) ) ) AND NOT ( TITLE-ABS-KEY ( children OR animal ) )**

**215 references**

**Database: EBM Reviews - Cochrane Database of Systematic Reviews** <2005 to September 5, 2019>, EBM Reviews - ACP Journal Club <1991 to August 2019>, EBM Reviews - Database of Abstracts of Reviews of Effects <1st Quarter 2016>, EBM Reviews - Cochrane Clinical Answers <August 2019>, EBM Reviews - Cochrane Central Register of Controlled Trials <August 2019>, EBM Reviews - Cochrane Methodology Register <3rd Quarter 2012>, EBM Reviews - Health Technology Assessment <4th Quarter 2016>, EBM Reviews - NHS Economic Evaluation Database <1st Quarter 2016>

Search Strategy:

--------------------------------------------------------------------------------

1 (critically ill or intensive care or critical illness or critical care).ti,ot,ab,kw,ct,sh,hw. (27839)

2 (beta adrenergic blocker or beta blocker*).ti,ot,ab,kw,ct,sh,hw. (7366)

3 landiolol.ti,ot,ab,kw,ct,sh,hw. (168)

4 esmolol.ti,ot,ab,kw,ct,sh,hw. (693)

5 labetalol.ti,ot,ab,kw,ct,sh,hw. (746)

6 bisoprolol.ti,ot,ab,kw,ct,sh,hw. (967)

7 metoprolol.ti,ot,ab,kw,ct,sh,hw. (3115)

8 or/2-7 (11296)

9 1 and 8 (168)

10 (RCT or randomized).ti,ot,ab,kw,ct,sh,hw. (816843)

11 9 and 10 (121)

12 children.ti,ot,ab,kw,ct,sh,hw. (97694)

13 animal.ti,ot,ab,kw,ct,sh,hw. (19429)

14 12 or 13 (116146)

15 11 not 14 (117) children and animals removed

16 remove duplicates from 15 (109)

***************************

Database: Ovid MEDLINE(R) and Epub Ahead of Print, In-Process, In-Data-Review & Other Non-Indexed Citations and Daily <1946 to March 04, 2021>

Search Strategy:

--------------------------------------------------------------------------------

1 (critically ill or intensive care or critical illness or critical care).mp. (255749)

2 exp Critical Care/ (59822)

3 exp Critical Illness/ (31131)

4 severely ill.mp. (4892)

5 sepsis.mp. or exp SEPSIS/ (187128)

6 septic shock.mp. or exp Shock, Septic/ (35867)

7 exp SHOCK/ or shock.mp. (247113)

8 circulatory failure.mp. (2221)

9 exp Intensive Care Units/ (88831)

10 emergency department.mp. or exp Emergency Service, Hospital/ (133875)

11 trauma.mp. (276977)

12 exp Trauma Centers/ (11123)

13 exp Multiple Trauma/ (13028)

14 severe injury.mp. (3693)

15 exp "Wounds and Injuries"/ or severely injured.mp. (924243)

16 severe burn.mp. (1916)

17 exp Burns/ (58317)

18 1 or 2 or 3 or 4 or 5 or 6 or 7 or 8 or 9 or 10 or 11 or 12 or 13 or 14 or 15 or 16 or 17 (1720488)

19 beta adrenergic blocker.mp. or exp Adrenergic beta-Antagonists/ (84985)

20 exp adrenergic beta-antagonists/ or exp adrenergic beta-1 receptor antagonists/ or exp adrenergic beta-2 receptor antagonists/ or exp adrenergic beta-3 receptor antagonists/ (84777)

21 landiolol.mp. (365)

22 esmolol.mp. (1487)

23 propranolol.mp. or exp PROPRANOLOL/ (45351)

24 bisoprolol.mp. or exp BISOPROLOL/ (1784)

25 metoprolol.mp. or exp METOPROLOL/ (8413)

26 19 or 20 or 21 or 22 or 23 or 24 or 25 or beta blocker*.mp. (111389)

27 exp Multiple Organ Failure/ or organ failure.mp. (28076)

28 organ dysfunction.mp. (13860)

29 recovery.mp. (504749)

30 27 or 28 or 29 (540635)

31 18 and 26 (3821)

32 limit 31 to ("all adult (19 plus years)" and clinical trial, all) (242)

33 exp MORTALITY/ or mortality.mp. (1323489)

34 outcome.mp. or exp Treatment Outcome/ or exp "OUTCOME ASSESSMENT (HEALTH CARE)"/ (2064721)

35 exp SURVIVAL/ or survival.mp. (1295737)

36 30 or 33 or 34 or 35 (4071629)

37 18 and 26 and 36 (1564)

38 limit 37 to ("all adult (19 plus years)" and clinical trial, all) (138)

39 limit 32 to yr="2019 -Current" (10)

40 (clinical or randomized or RCT).af. (6438626)

41 31 and 40 (1558)

42 (adult* or aged or elderly).af. (8346473)

43 41 and 42 (867)

44 limit 43 to yr="2019 -Current" (83)

45 (201909* or 201910* or 201911* or 201912*).ed. (349829)

46 limit 43 to yr="2020 -Current" (48)

47 43 and 45 (6)

48 46 or 47 (54)

***************************

Database: EBM Reviews - Cochrane Database of Systematic Reviews <2005 to March 3, 2021>, EBM Reviews - ACP Journal Club <1991 to February 2021>, EBM Reviews - Database of Abstracts of Reviews of Effects <1st Quarter 2016>, EBM Reviews - Cochrane Clinical Answers <February 2021>, EBM Reviews - Cochrane Central Register of Controlled Trials <February 2021>, EBM Reviews - Cochrane Methodology Register <3rd Quarter 2012>, EBM Reviews - Health Technology Assessment <4th Quarter 2016>, EBM Reviews - NHS Economic Evaluation Database <1st Quarter 2016>

Search Strategy:

--------------------------------------------------------------------------------

1 (critically ill or intensive care or critical illness or critical care).ti,ot,ab,kw,ct,sh,hw. (33417)

2 (beta adrenergic blocker or beta blocker*).ti,ot,ab,kw,ct,sh,hw. (7902)

3 landiolol.ti,ot,ab,kw,ct,sh,hw. (191)

4 esmolol.ti,ot,ab,kw,ct,sh,hw. (784)

5 labetalol.ti,ot,ab,kw,ct,sh,hw. (840)

6 bisoprolol.ti,ot,ab,kw,ct,sh,hw. (1048)

7 metoprolol.ti,ot,ab,kw,ct,sh,hw. (3245)

8 or/2-7 (12131)

9 1 and 8 (202)

10 (RCT or randomized).ti,ot,ab,kw,ct,sh,hw. (981735)

11 9 and 10 (146)

12 children.ti,ot,ab,kw,ct,sh,hw. (113401)

13 animal.ti,ot,ab,kw,ct,sh,hw. (21896)

14 12 or 13 (134124)

15 11 not 14 (139)

16 remove duplicates from 15 (131)

17 limit 16 to yr="2019 -Current" [Limit not valid in DARE; records were retained] (20)

***************************

SCOPUS UPDATE March 3, 2021

( ( TITLE-ABS-KEY ( "critically ill"  OR  "intensive care"  OR  "critical illness"  OR  "critical care" ) )  AND  ( ( TITLE-ABS-KEY ( "beta adrenergic blocker"  OR  "beta blocker" )  OR  TITLE-ABS-KEY ( esmolol  OR  landiolol  OR  bisoprolol  OR  metoprolol  OR  labetalol ) ) )  AND  ( TITLE-ABS-KEY ( randomized  OR  rct ) ) )  AND NOT  ( TITLE-ABS-KEY ( children  OR  animal ) )
